# Supplementary material for: A last-in first-out stack data structure implemented in DNA
Source: Nat Commun. 2021 Aug 11;12:4861. doi: 10.1038/s41467-021-25023-6 (PMC8358042; doi:10.1038/s41467-021-25023-6)
Supplement: Supplementary file 1 — Supplementary Information New [file 41467_2021_25023_MOESM1_ESM.pdf]

Supplementary Information

A Last-In First-Out Stack Data Structure Implemented in DNA

Annunziata Lopiccolo[1†], Ben Shirt-Ediss[1†], Emanuela Torelli[1], Abimbola Feyisara Adedeji Olulana[2,3], Matteo Castronovo[2,3], Harold Fellermann[1⋆] and Natalio Krasnogor[1⋆]

[†]These authors contributed equally: Annunziata Lopiccolo and Ben Shirt-Ediss

[1] Interdisciplinary Computing and Complex Biosystems Research Group, School of Computing, Newcastle University, Newcastle-upon-Tyne, NE4 5TG, UK

[2] Food Colloids and Bioprocessing Group, School of Food Science and Nutrition, University of Leeds, Leeds LS2 9JT, UK

[3] Regional Referral Centre for Rare Diseases, Azienda Sanitaria Universitaria Integrata di Udine, 33100 Udine, Italy

[*]To whom correspondence should be addressed: harold.fellermann@newcastle.ac.uk, natalio.krasnogor@newcastle.ac.uk.

Contents

Supplementary Note 1
DNA Sequences 3

Supplementary Note 2
Predicted Secondary Structures of DNA Strands 3

Supplementary Note 3
Detailed Lane Reaction Sequences 5

Supplementary Note 4
Stack Assembly in Washing and No Washing Conditions 11

Supplementary Note 5
Persistence of Stack Nanostructures on Freeze-Thaw 12

Supplementary Note 6
Additional AFM Results 13

Supplementary Note 7
Electrophoretic Mobility of Linear Signal Stack Assemblies 14

Supplementary Note 8
Determination of Hybridisation Rate Constants by UV Absorbance 16

Supplementary Note 9
Kinetic Model of Stack Chemistry 18

Supplementary Note 10
Expanded Model Results 37

Supplementary Note 11
Oligonucleotide Synthesis Purity 56

Supplementary Note 1 DNA Sequences

Synthetic DNA sequences in Supplementary Table 1 below were designed using evolutionary optimisation [1].

Strand		Length	Domains	Primary Sequence 5'-3'
Start	s	50nt	Al*	CACACTATTTCCCTTCTACCCGCCCTATCTCATCTCTCATC TCATCTTAA
Push	p	56nt	A*BC	ATAGGGCGGGTAGAAGGGAAATAGTGTGATCCAGTTATT ATAGTTTTGAAGCGTAT
Write	w	56nt	AC*B*	CACACTATTTCCCTTCTACCCGCCCTATATACGCTTCAAA ACTATAATAACTGGAT
Write X	X	107nt	AC*vfXgv*B*	CACACTATTTCCCTTCTACCCGCCCTATATACGCTTCAAA ACGTGCAACAGTCGACTAAAAAAAACTGCTTGTATCTGCC CATACTGTTGCACTATAATAACTGGAT
Write Y	Y	137nt	AC*whYiw*B*	CACACTATTTCCCTTCTACCCGCCCTATATACGCTTCAAA ACGCCTTGCGTGCGCCTACCTCGAAATTCACCACCCCCA CCTCTCTTTTATATCCCACATTTCGAGGTAGGCGCACGCA AGGCTATAATAACTGGAT
Read	r	56nt	BCA*	ATCCAGTTATTATAGTTTTGAAGCGTATATAGGGCGGGTA GAAGGGAAATAGTGTG
Pop	q	56nt	C*B*A	ATACGCTTCAAAACTATAATAACTGGATCACACTATTTCCC TTCTACCCGCCCTAT
Linker	k	33nt	ml	GAGAGAGATGATTAAGATGAGATGAGAGATGAG
Releaser	z	33nt	l*m*	CTCATCTCTCATCTCATCTTAATCATCTCTCTC

Supplementary Table 1: Nine ssDNA strands used to realise the stack data structure *in vitro*.

Supplementary Note 2 Predicted Secondary Structures of DNA Strands

Minimum free energy (MFE) structures in Supplementary Table 2 below are calculated using ViennaRNA v2.4.1 with Mathews 2004 DNA parameters at 21C.

Strand		Domains / MFE Structure	Strand		Domains / MFE Structure
Start	s	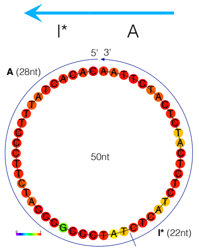	Read	r	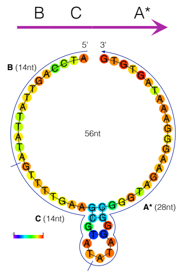
Push	p	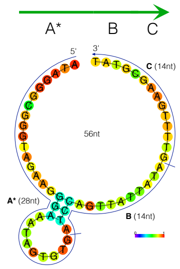	Pop	q	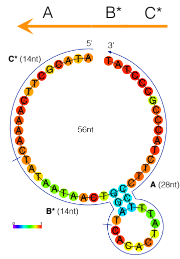
Write	w	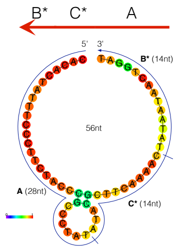	Linker	k	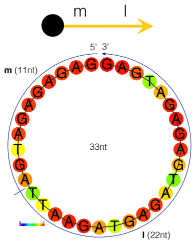
Write X	X	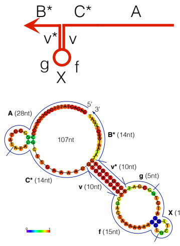	Releaser	z	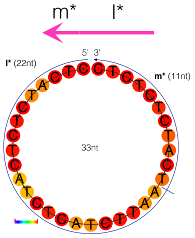
Write Y	Y	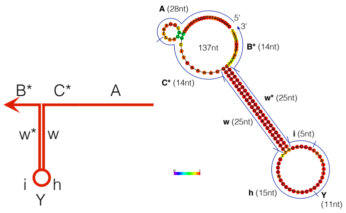			

Supplementary Table 2: Domain-level representations and MFE structures of DNA complexes. Colour denotes base pairing probability. Zoom for detail.

Supplementary Note 3 Detailed Lane Reaction Sequences

Supplementary Note 3.1 10% PAGE

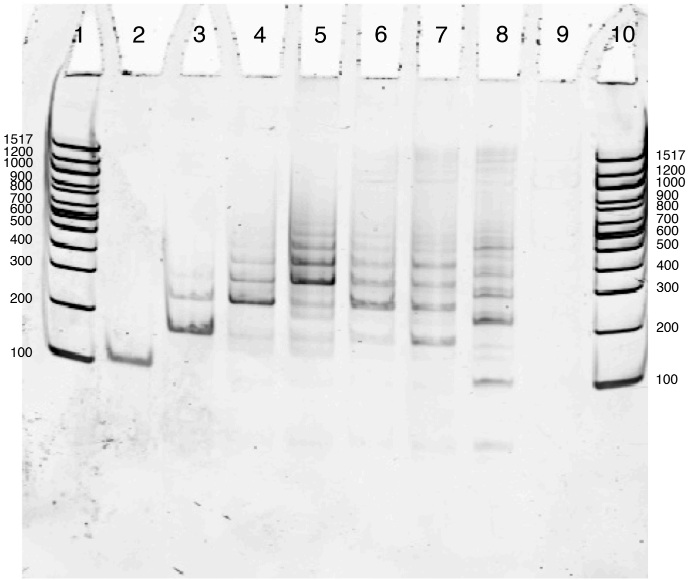

Supplementary Figure 1: Recording then popping 3 *write* signals (Figure 2a main paper). 100bp DNA ladder (NEB). Ladder molecular weight markers in base pairs.

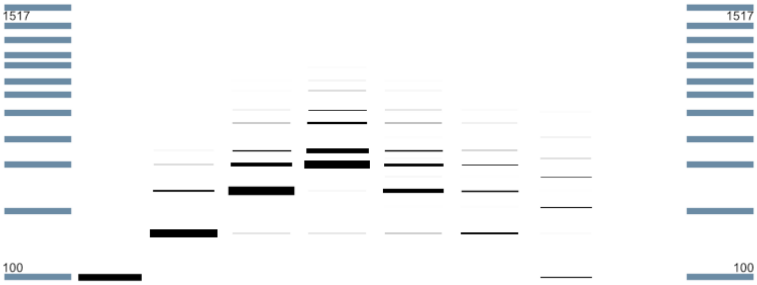

Supplementary Figure 2: Simulation of recording then popping 3 *write* signals (Figure 2a main paper).

Lane	Reaction Sequence	Target Stack
1	NEB 100bp DNA ladder	
2	k - ● - s - ● - p - ● - z - ○ - PAGE	sp
3	k - ● - s - ● - p - ● - w - ● - z - ○ - PAGE	spw
4	k - ● - s - ● - p - ● - w - ● - p - ● - w - ● - z - ○ - PAGE	spwpw
5	k - ● - s - ● - p - ● - w - ● - p - ● - w - ● - p - ● - w - ● - z - ○ - PAGE	spwpwpw
6	k - ● - s - ● - p - ● - w - ● - p - ● - w - ● - p - ● - w - ● - r - ● - q - ● - z - ○ - PAGE	spwpw
7	k - ● - s - ● - p - ● - w - ● - p - ● - w - ● - p - ● - w - ● - r - ● - q - ● - r - ● - q - ● - z - ○ - PAGE	spw
8	k - ● - s - ● - p - ● - w - ● - p - ● - w - ● - p - ● - w - ● - r - ● - q - ● - r - ● - q - ● - r - ● - z - ○ - PAGE	sp
10	NEB 100bp DNA ladder	
	● = wait 30 minutes then perform washing step. ○ = wait 30 minutes. PAGE = PAGE analysis of supernatant.	
	Linker (k) and *releaser* (z) concentrations nominally 200nM. All other strand concentrations nominally 300nM.	

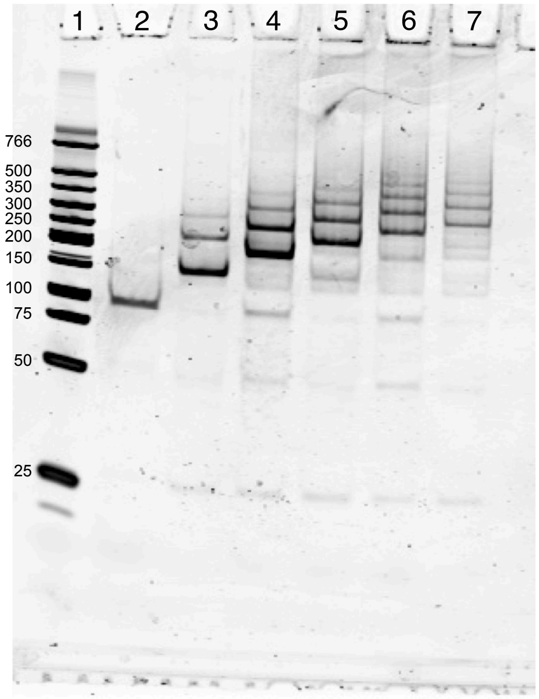

Supplementary Figure 3: Recording 3 *write* signals (Figure 2b main paper). Low molecular weight DNA ladder (NEB). Ladder molecular weight markers in base pairs.

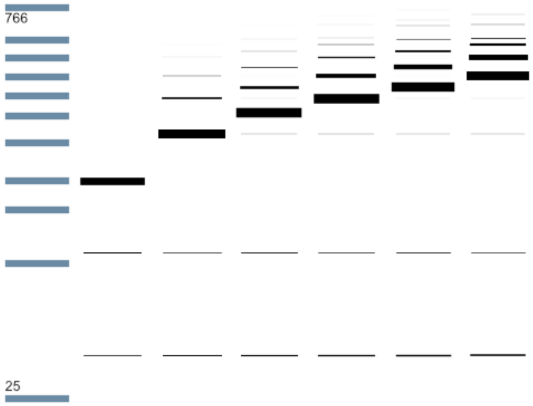

Supplementary Figure 4: Simulation of recording 3 *write* signals (Figure 2b main paper).

Lane	Reaction Sequence	Target Stack
1	NEB low molecular weight ladder	
2	k - ● - s - ● - p - ● - z - ○ - PAGE	sp
3	k - ● - s - ● - p - ● - w - ● - z - ○ - PAGE	spw
4	k - ● - s - ● - p - ● - w - ● - p - ● - z - ○ - PAGE	spwp
5	k - ● - s - ● - p - ● - w - ● - p - ● - w - ● - z - ○ - PAGE	spwpw
6	k - ● - s - ● - p - ● - w - ● - p - ● - w - ● - p - ● - z - ○ - PAGE	spwpwp
7	k - ● - s - ● - p - ● - w - ● - p - ● - w - ● - p - ● - w - ● - z - ○ - PAGE	spwpwpw
	● = wait 30 minutes then perform washing step. ○ = wait 30 minutes. PAGE = PAGE analysis of supernatant.	
	Linker (k) and *releaser* (z) concentrations nominally 200nM. All other strand concentrations nominally 300nM.	

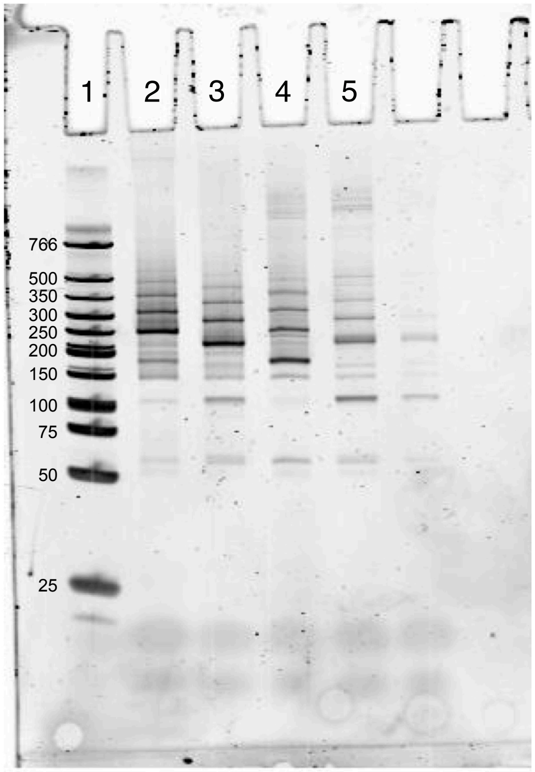

Supplementary Figure 5: Popping from 2 *write* signals (Figure 2c main paper). Low molecular weight DNA ladder (NEB). Ladder molecular weight markers in base pairs.

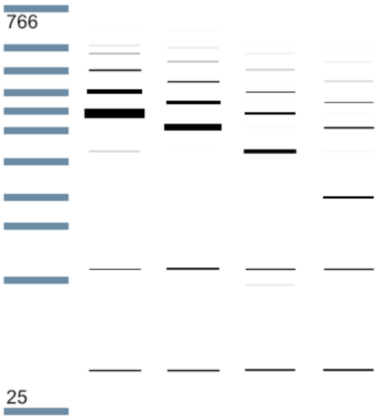

Supplementary Figure 6: Simulation of popping from 2 *write* signals (Figure 2c main paper).

Lane	Reaction Sequence	Target Stack
1	NEB low molecular weight ladder	
2	k - ● - s - ● - p - ● - w - ● - p - ● - w - ● - z - ○ - PAGE	spwpw
3	k - ● - s - ● - p - ● - w - ● - p - ● - w - ● - r - ● - z - ○ - PAGE	spwp
4	k - ● - s - ● - p - ● - w - ● - p - ● - w - ● - r - ● - q - ● - z - ○ - PAGE	spw
5	k - ● - s - ● - p - ● - w - ● - p - ● - w - ● - r - ● - q - ● - r - ● - z - ○ - PAGE	sp
	● = wait 30 minutes then perform washing step. ○ = wait 30 minutes. PAGE = PAGE analysis of supernatant.	
	Linker (k) and *releaser* (z) concentrations nominally 200nM. All other strand concentrations nominally 300nM.	

Supplementary Note 3.2 Bioanalyzer

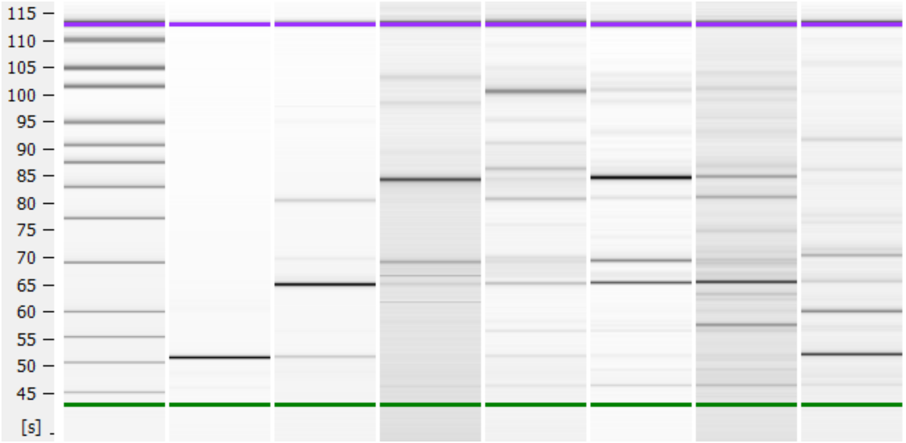

Supplementary Figure 7: Recording and then popping signals *X, Y, X* (Figure 4a main paper).

Lane	Reaction Sequence	Target Stack
1	Bioanalyzer migration time ladder	
2	k - ● - s - ● - p - ● - z - ○ - Bioanalyzer	sp
3	k - ● - s - ● - p - ● - X - ● - z - ○ - Bioanalyzer	spX
4	k - ● - s - ● - p - ● - X - ● - p - ● - Y - ● - z - ○ - Bioanalyzer	spXpY
5	k - ● - s - ● - p - ● - X - ● - p - ● - Y - ● - p - ●- X - ●- z - ○ - Bioanalyzer	spXpYpX
6	k - ● - s - ● - p - ● - X - ● - p - ● - Y - ● - p - ●- X - ●- r - ●- q - ● - z - ○ - Bioanalyzer	spXpY
7	k - ● - s - ● - p - ● - X - ● - p - ● - Y - ● - p - ●- X - ●- r - ●- q - ● - r - ●- q - ●- z - ○ - Bioanalyzer	spX
8	k - ● - s - ● - p - ● - X - ● - p - ● - Y - ● - p - ●- X - ●- r - ●- q - ● - r - ●- q - ●- r - ●- z - ○ - Bioanalyzer	sp
	● = wait 30 minutes then perform washing step. ○ = wait 30 minutes. Bioanalyzer = Bioanalyzer analysis of supernatant.	
	Linker (k) and *releaser* (z) concentrations nominally 200nM. All other strand concentrations nominally 300nM, except *read* at 50nM.	

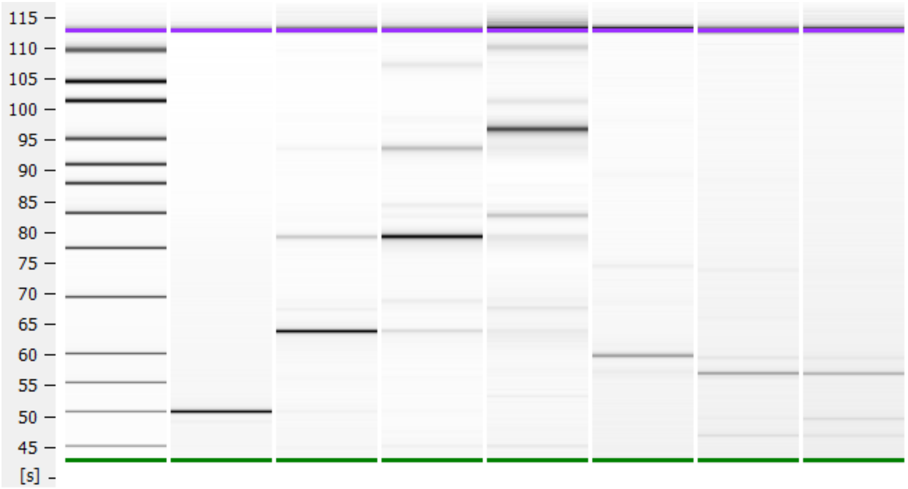

Supplementary Figure 8: Recording signals *X*, *X*, *Y* and then examining supernatant after each pop (Figure 4c main paper).

Lane	Reaction Sequence	Target Stack
1	Bioanalyzer migration time ladder	
2	k - ● - s - ● - p - ● - z - ○ - Bioanalyzer	sp
3	k - ● - s - ● - p - ● - X - ● - z - ○ - Bioanalyzer	spX
4	k - ● - s - ● - p - ● - X - ● - p - ● - X - ● - z - ○ - Bioanalyzer	spXpX
5	k - ● - s - ● - p - ● - X - ● - p - ● - X - ● - p - ●- Y - ●- z - ○ - Bioanalyzer	spXpXpY
6	k - ● - s - ● - p - ● - X - ● - p - ● - X - ● - p - ●- Y - ●- r - ○ - Bioanalyzer	Yr in supernatant
7	k - ● - s - ● - p - ● - X - ● - p - ● - X - ● - p - ●- Y - ●- r - ●- q - ● - r - ○ - Bioanalyzer	Xr in supernatant
8	k - ● - s - ● - p - ● - X - ● - p - ● - X - ● - p - ●- Y - ●- r - ●- q - ● - r - ●- q - ●- r - ○ - Bioanalyzer	Xr in supernatant
	● = wait 30 minutes then perform washing step. ○ = wait 30 minutes. Bioanalyzer = Bioanalyzer analysis of supernatant.	
	Linker (k) and *releaser* (z) concentrations nominally 200nM. All other strand concentrations nominally 300nM, except *read* at 50nM.	

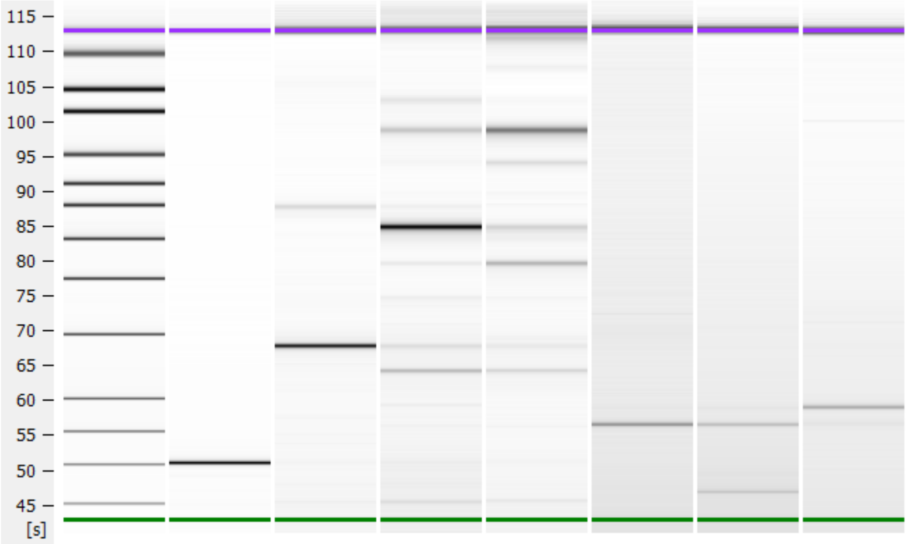

Supplementary Figure 9: Recording signals *Y, X, X* and then examining supernatant after each pop (Figure 4d main paper).

Lane	Reaction Sequence	Target Stack
1	Bioanalyzer migration time ladder	
2	k - ● - s - ● - p - ● - z - ○ - Bioanalyzer	sp
3	k - ● - s - ● - p - ● - Y - ● - z - ○ - Bioanalyzer	spY
4	k - ● - s - ● - p - ● - Y - ● - p - ● - X - ● - z - ○ - Bioanalyzer	spYpX
5	k - ● - s - ● - p - ● - Y - ● - p - ● - X - ● - p - ●- X - ●- z - ○ - Bioanalyzer	spYpXpX
6	k - ● - s - ● - p - ● - Y - ● - p - ● - X - ● - p - ●- X - ●- r - ○ - Bioanalyzer	Xr in supernatant
7	k - ● - s - ● - p - ● - Y - ● - p - ● - X - ● - p - ●- X - ●- r - ●- q - ● - r - ○ - Bioanalyzer	Xr in supernatant
8	k - ● - s - ● - p - ● - Y - ● - p - ● - X - ● - p - ●- X - ●- r - ●- q - ● - r - ●- q - ●- r - ○ - Bioanalyzer	Yr in supernatant
	● = wait 30 minutes then perform washing step. ○ = wait 30 minutes. Bioanalyzer = Bioanalyzer analysis of supernatant.	
	Linker (k) and *releaser* (z) concentrations nominally 200nM. All other strand concentrations nominally 300nM, except *read* at 50nM.	

Supplementary Note 4 Stack Assembly in Washing and No Washing Conditions

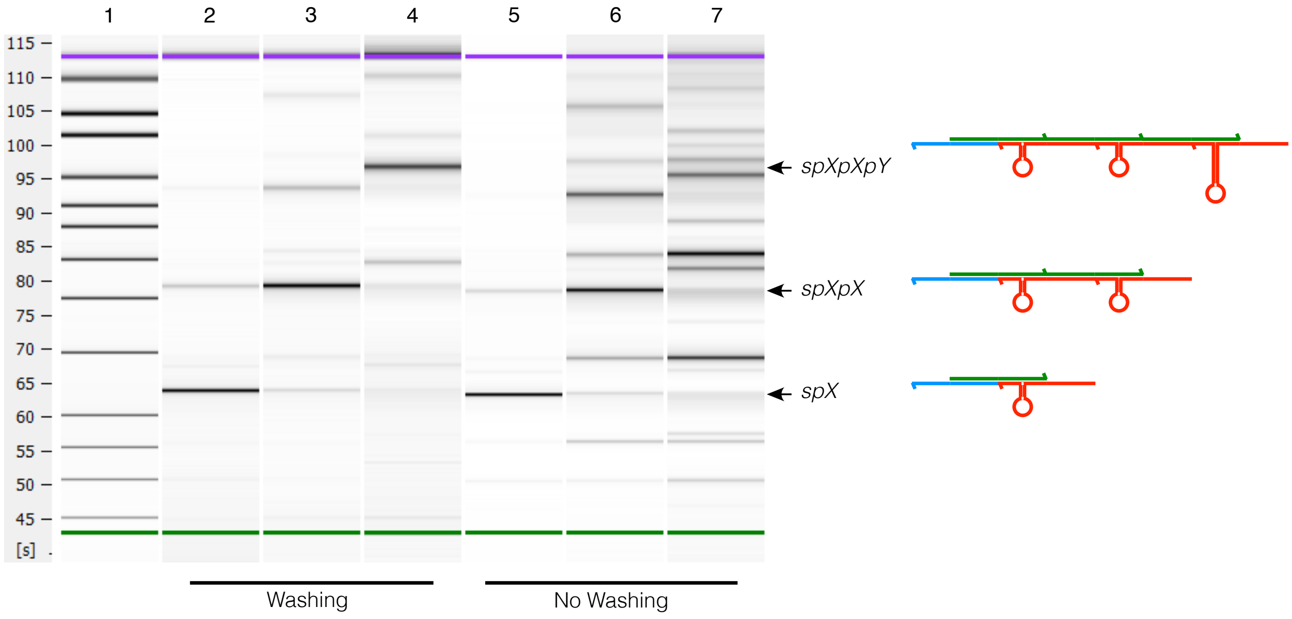

Supplementary Figure 10: Washing steps decrease side reactions. Recording three signals *X*, *X*, *Y* on the stack with washing steps (lanes 2-4) compared to the same assembly without washing steps (lanes 5-7). Due to reaction irreversibility and polymerisation, operation of the system under no washing conditions leads to an extreme sensitivity on the pipetted concentration of strands. Capillary electrophoresis performed by Agilent 2100 Bioanalyzer. Experiment was repeated independently 3 times.

Lane	Reaction Sequence
1	Bioanalyzer migration time ladder
2	k - ● - s - ● - p - ● - X - ● - z - ○ - Bioanalyzer
3	k - ● - s - ● - p - ● - X - ● - p - ● - X - ● - z - ○ - Bioanalyzer
4	k - ● - s - ● - p - ● - X - ● - p - ● - X - ● - p - ● - Y - ● - z - ○ - Bioanalyzer
5	s - ○ - p - ○ - X - ○ - Bioanalyzer
6	s - ○ - p - ○ - X - ○ - p - ○ - X - ○ - Bioanalyzer
7	s - ○ - p - ○ - X - ○ - p - ○ - X - ○ - p - ○ - Y - ○ - Bioanalyzer
	● = wait 30 minutes then perform washing step. ○ = wait 30 minutes. Bioanalyzer = Bioanalyzer analysis of supernatant.
	Linker (k) and *releaser* (z) concentrations nominally 200nM. All other strand concentrations nominally 300nM.

Supplementary Note 5 Persistence of Stack Nanostructures on Freeze-Thaw

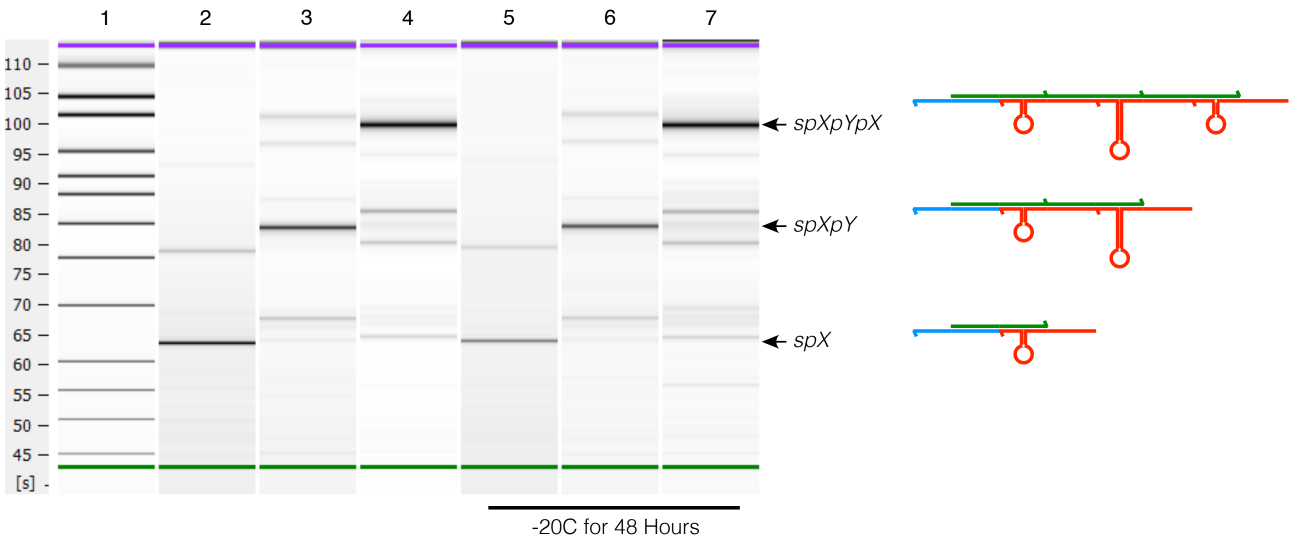

Supplementary Figure 11: Freezing does not affect nanostructure integrity. Stack assembly (*X*,*Y*,*X*) at room temperature (lanes 2-4). Subsequent freezing of reaction solutions at -20C for 48h, and then thawing to 4C followed by electrophoresis (lanes 5-7). Capillary electrophoresis performed by Agilent 2100 Bioanalyzer. Experiment was repeated independently 3 times. This result suggests that experiments involving many push and pop operations could effectively be split over consecutive days by freezing samples overnight.

Lane	Reaction Sequence
1	Bioanalyzer migration time ladder
2	k - ● - s - ● - p - ● - X - ● - z - ○ - Bioanalyzer
3	k - ● - s - ● - p - ● - X - ● - p - ● - Y - ● - z - ○ - Bioanalyzer
4	k - ● - s - ● - p - ● - X - ● - p - ● - Y - ● - p - ● - X - ● - z - ○ - Bioanalyzer
	● = wait 30 minutes then perform washing step. ○ = wait 30 minutes. Bioanalyzer = Bioanalyzer analysis of supernatant.
	Linker (k) and *releaser* (z) concentrations nominally 200nM. All other strand concentrations nominally 300nM.

Supplementary Note 6   Additional AFM Results

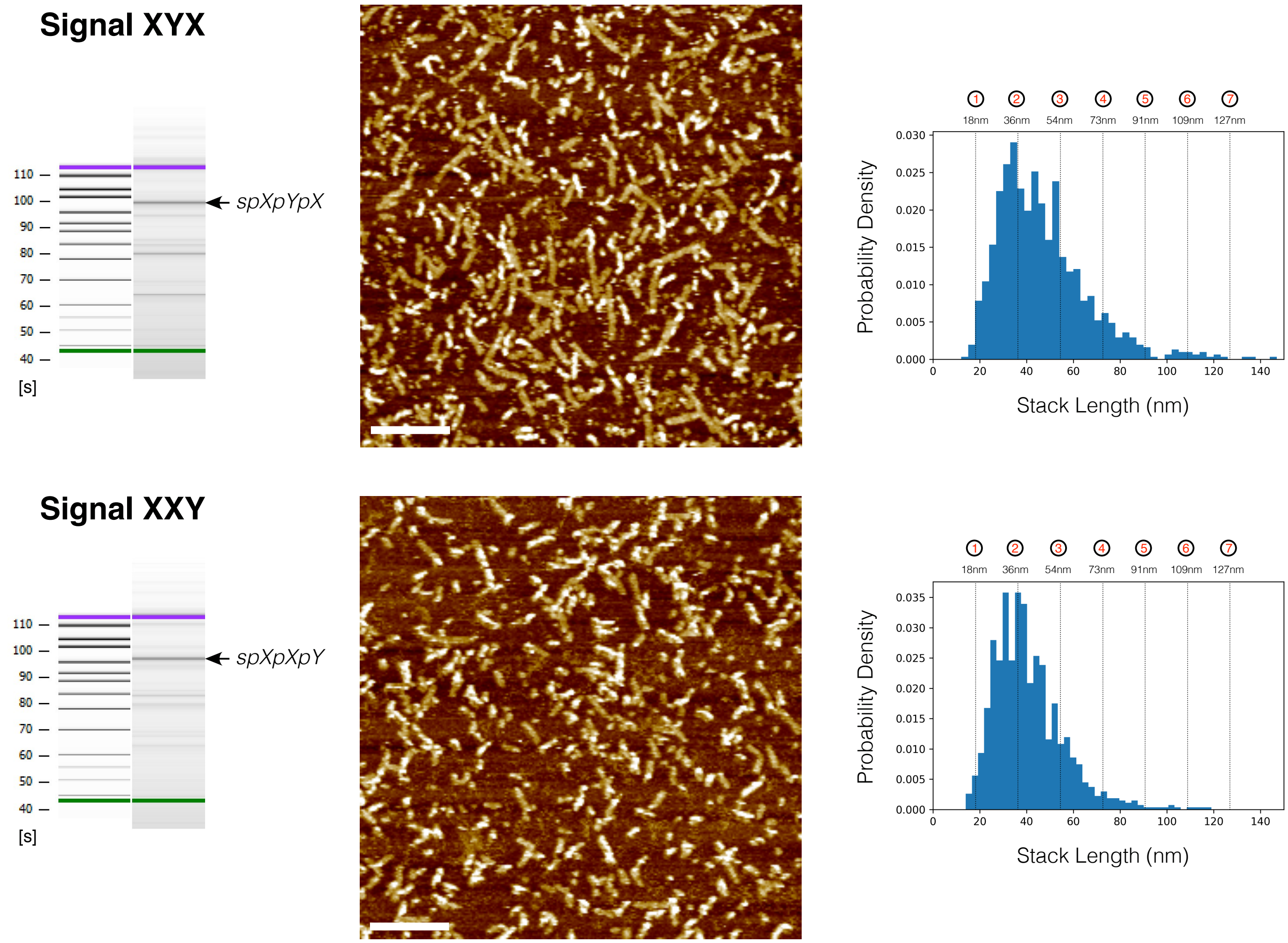

Supplementary Figure 12: AFM micrographs of stacks assembled to record signals in order *X*, *Y*, *X* (top) and *X*, *X*, *Y* (bottom). Scale bar 200nm. For each of the stack size distributions (right) 1022 measurements were manually curated from AFM micrographs. XYX was assembled by reaction sequence k - ● - s - ● - p - ● - X - ● - p - ● - Y - ● - p - ● - X - ● - z - ○ - (supernatant at -20C for transport) - AFM and XXY was assembled by k - ● - s - ● - p - ● - X - ● - p - ● - X - ● - p - ● - Y - ● - z - ○ - (supernatant at -20C for transport) - AFM. *Linker* (k) and *releaser* (z) concentrations nominally 200nM. All other strand concentrations nominally 300nM. Wait times 30 minutes.

Supplementary Note 7 Electrophoretic Mobility of Linear Signal Stack Assemblies

Linear stack structures (with no hairpin loops) were found to have predictable electrophoretic mobility in 10% PAGE, allowing accurate identification of gel bands (Supplementary Figure 13). This was a crucial factor permitting experiments to be matched with simulation model data.

Supplementary Figure 13 shows how linear stack structures migrate more slowly (blue line) than dsDNA helix products of the same molecular weight (black line) when run in 10% polyacrylamide gel. The approximate relationship between running bp and total number of nucleotide bases in a stack complexes of 2 or more strands was fitted via least squares as:

$$\mathrm{bp}_{\mathrm{running}} = 0.7\mathrm{nt} + 35 \tag{1}$$

The reduced electrophoretic mobility of linear stack structures is likely due to (i) the nicked helix backbone making the complex more flexible, and (ii) the 28bp single stranded overhangs which will assume a random coil shape (since any secondary structure here is designed out). Slower migration of duplexes with ssDNA overhangs is consistent with previous studies [2].

We confirmed that single stranded species *push* and *write* (56nt) migrated at approximately the same running bp as same length double stranded species *push-pop* and *read-write* (56bp). The latter two species migrated exactly with the dsDNA ladder, as expected.

Species *start* (50nt) migrated at the lowest running bp (approx. 49bp). Additionally, *start* demonstrated exceptionally poor intercalation of SYBR Gold dye as compared to the other DNA strands.

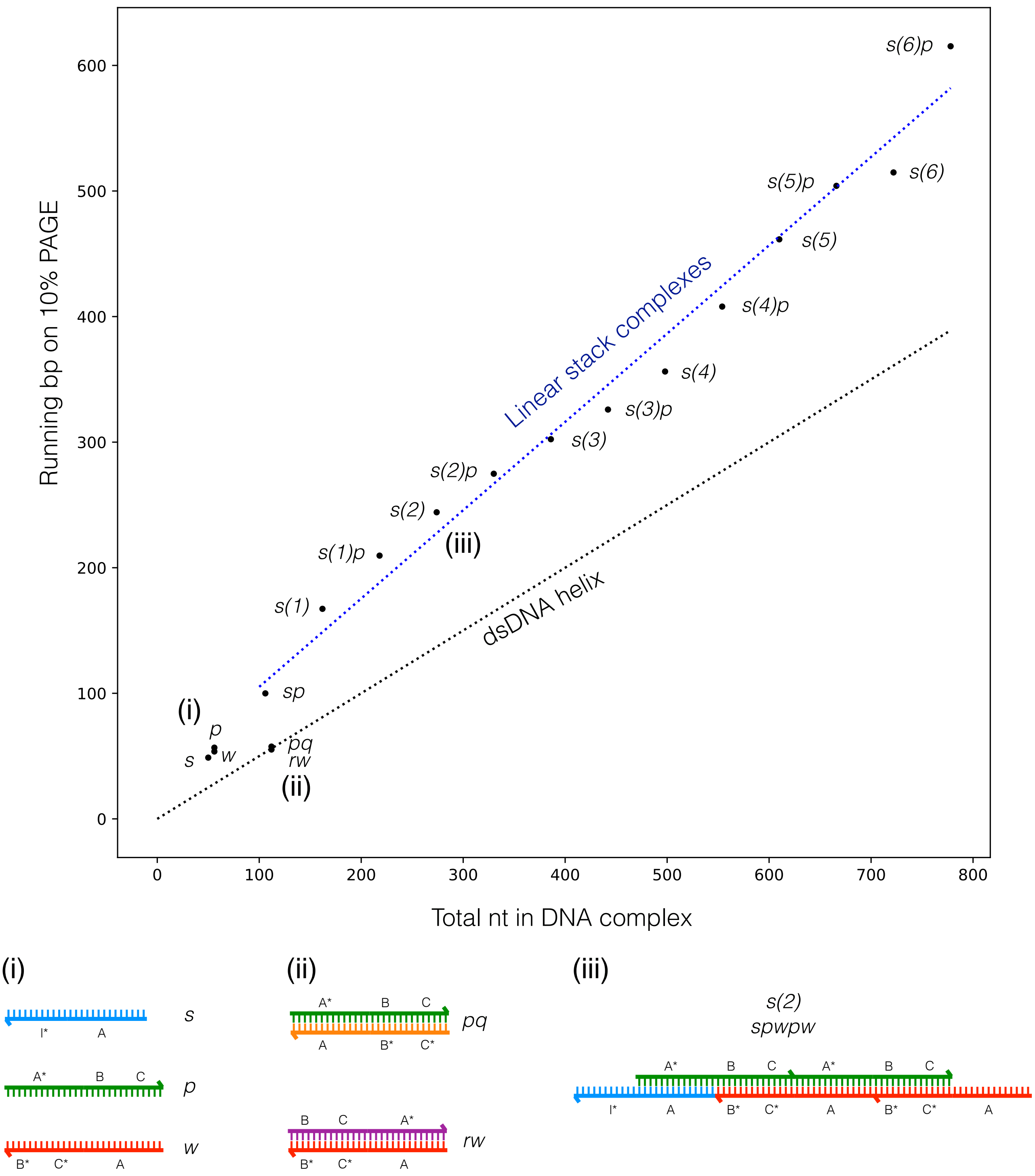

Supplementary Figure 13: Electrophoretic mobility of stack species in 10% polyacrylamide gel. (i) Single strands, (ii) double stranded helix products and (iii) linear stack structures. Species *s(1)* is shorthand for *spw*, *s(1)p* for *spwp*, *s(2)* for *spwpw* etc. All running bp values collected and averaged from 32 polyacrylamide gel images of varied experiments provided in the following Zenodo data set doi:10.5281/zenodo.5060760.

Supplementary Note 8 Determination of Hybridisation Rate Constants by UV Absorbance

Hybridisation rate constants k_A, k_{BC} and k_{ABC} were approximately determined by following the UV absorbance at 260nm of reactions *start+push*, *write+p-glow* and *push+pop*, respectively. Strand concentrations used were 1μM. *P-glow* was a special strand designed to hybridise only on the B*C* domain of *write* such that polymerisation did not occur (the Cy5 modification was not used). Purity of strands was confirmed by denaturing PAGE.

Strand	Length	Domains	Primary Sequence 5'-3'
p-glow	56nt	**Cy5**-BCL	**Cy5**-ATCCAGTTATTATAGTTTTGAAGCGTATCCTCTCCTCC ATTTATCCTCCTCCATCC

Absorbance experiments were selected which featured a decay trajectory to a stable absorbance value A_∞ after mixing.

First, strand concentrations at the initial instant of mixing were calculated. The strand initially in the cuvette, i, had its concentration at mixing determined by the Beer-Lambert law:

$$[i]_0 = \frac{< A_i >}{\epsilon_i l} \left(\frac{V_i}{V_{\text{final}}} \right) \tag{2}$$

where $< A_i >$ is the absorbance value at 260nm of strand *i* alone, averaged over 3 minutes, ϵ_i is the nearest neighbour extinction coefficient of i (calculated using IDT Oligoanalyzer) and l is the path length of the sample. The dilution term V_i/V_{final} reflects that the concentration of the initial strand in volume V_i decreases when the second strand is added and the volume increases to V_{final}. In our experiments $V_i/V_{final} = 0.893$.

The concentration of the added strand x at the instant of mixing could not be obtained experimentally because of the pipette obstructing the light path. Instead, it was deduced in a reverse manner by using the stable absorbance value when the reaction had completed A_∞, and using the concentration of the first strand $[i]_0$:

$$[x]_0 = \min \left\{ \frac{A_\infty - \epsilon_i [i]_0}{\epsilon_{\text{duplex}} - \epsilon_i}, \frac{A_\infty - (\epsilon_{\text{duplex}} - \epsilon_x)[i]_0}{\epsilon_x} \right\} \tag{3}$$

where ϵ_x is the nearest neighbour extinction coefficient of the added strand. Equation 3 is derived by considering that the final absorbance A_∞ when $[i]_0 \geq [x]_0$ is given by:

$$A_\infty^{i \geq x} = \epsilon_{\text{duplex}} l [x]_0 + \epsilon_i l ([i]_0 - [x]_0)$$

and when $[x]_0 \geq [i]_0$, A_∞ is given by:

$$A_\infty^{x \geq i} = \epsilon_{\text{duplex}} l [i]_0 + \epsilon_x l \left([x]_0 - [i]_0 \right)$$

Note that Equation 3 above assumes that the hybridisation reaction completes to 100% at equilibrium, which may not be strictly true if transient bind-states of the strands can kinetically block hybridisation.

The extinction coefficient for duplexed DNA, ϵ_{duplex} was an important parameter to calculate. For the blunt-ended *push-pop* duplex, we used the hypochromicity formula

$$\epsilon_{\text{duplex}} = (\epsilon_1 + \epsilon_2) \times (1 - h) \tag{4}$$

where ϵ_1 and ϵ_2 are the extinction coefficients of the sense and anti-sense strands of the duplex when in isolation and hypochromicity factor $h = (0.059 \times fGC) + (0.287 \times fAT)$. Parameters fAT and fGC are the respective fractions of AT and GC base pairs in the duplex.

For duplexes with dangling ends (*start-push* and *write- p-glow*), we approximated the overall extinction coefficient of the complex to be that of the dsDNA duplex section, plus the nearest neighbour extinction coefficients of the ssDNA dangles (i.e. essentially considering the dangles as independent molecules in solution):

$$\epsilon_{\text{duplex+overhangs}} = \epsilon_{\text{duplex}} + \epsilon_{\text{overhang1}} + \epsilon_{\text{overhang2}} \tag{5}$$

Once initial strand concentrations $[i]_0$ and $[x]_0$ were calculated by (2) and (3), the ODE for an irreversible bi-molecular reaction $i + x \xrightarrow{k} \text{duplex}$ was numerically integrated:

$$\frac{d[i]}{dt} = \frac{d[x]}{dt} = -\frac{d[\text{duplex}]}{dt} = -k[i][x] \tag{6}$$

converting concentrations at each time point to a nett absorbance value using again the Beer-Lambert law:

$$A_{\text{model}} = \epsilon_i l[i] + \epsilon_x l[x] + \epsilon_{\text{duplex}} l[\text{duplex}] \tag{7}$$

Rate constant parameter k was obtained by least-squares fitting the absorbance trajectory from experiment to A_{model}. Note: This model did *not* assume that the reactant strands were equimolar. The phase where the solution was hand mixed by pipette was not included in the experimental trajectory data. Rate constants were calculated for individual trajectories and then averaged. Dangling ends on *start-push* and *write- p-glow* duplexes were assumed not to influence the hybridisation rate of the central section.

Rate constants were found to be approximately $k_A \approx k_{BC} \approx 3 \times 10^4 M^{-1} s^{-1}$ and $k_{ABC} \approx 2.5 \times 10^5 M^{-1} s^{-1}$ at 25 °C. These estimates for k_A and k_{BC} agree in magnitude with recent studies of hybridisation kinetics of 20bp duplexes at 25 °C [3]. Further, $k_{ABC} > k_A \approx k_{BC}$ is consistent with reports that longer complementary strands hybridise faster than shorter complementary strands due to the presence of more base pairs to initiate first contact [4, 5].

If the reaction start point was considered to be slightly later than the first instant of pipetting (+ 5 seconds), this did not change the fitted rate constant values significantly.

Supplementary Note 9 Kinetic Model of Stack Chemistry

Simulation code and documentation is available at:<br>https://dnastack.readthedocs.io

A rule-based model of the stack chemistry was simulated as a stochastic process using `stocal` (https://github.com/harfel/stocal), a framework for rule-based Gillespie algorithm simulations particularly suited to polymerisation chemistries.

DNA chemical reactions were modelled using bi-molecular mass action kinetics. DNA hybridisation and strand displacement reactions were assumed to follow a simplified two-part model that neglected intermediate binding states on the way to full domain hybridisation (although such intermediate states may have been present in the experimental system and could have led to less than 100% reaction yields at equilibrium). The reaction solution was considered to be well-mixed. Washing events were modelled as immediate discontinuous jumps in the system state. Reactions were considered only to occur when the next incoming strand was added, not during the washing process itself, and thus the system volume was regarded as constant. On each washing event, (i) all stack species tethered specifically to sepharose beads had their particle number decreased by μ percent to simulate bead loss and (ii) a volume fraction ϕ of DNA species in the supernatant solution (i.e. not specifically tethered to beads) were also considered to survive the washing step and to carry through to the proceeding reaction phase. This latter effect modelled possible non-specific binding of DNA species to sepharose beads.

Supplementary Note 9.1 Reaction Rules

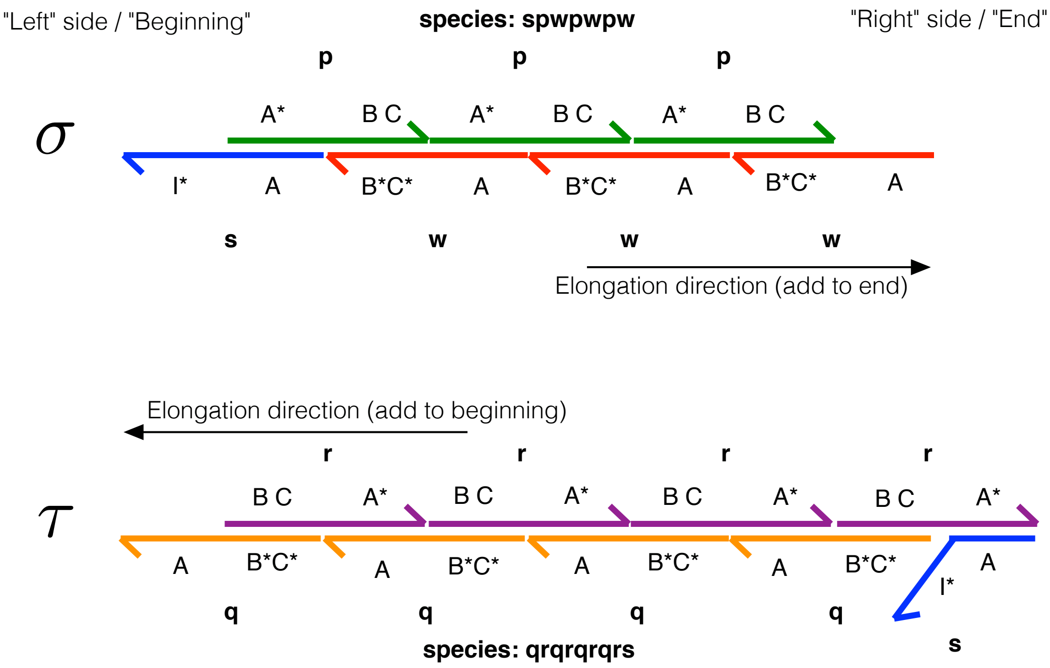

Supplementary Figure 14: Two types of polymers in the DNA stack chemistry. Only the σ polymer is a stack able to store different signal types (e.g. X, Y or w). The σ polymer pictured here contains linear *write* signals, which are the same as X and Y without a hairpin loop.

In total 20 reaction rules were considered for the DNA stack chemistry (Supplementary Table 3). Two types of polymer are able to form in the chemistry - σ stack polymers able to store X and Y signals and

also linear τ polymers made of alternating *read-pop* strands (Supplementary Figure 14). τ polymers appear when popping information, and are an unavoidable consequence of the design: *read* is the full complement of *write* w (or the linear backbone of signals *X* or *Y*), *pop* is the full complement of *push* and the full complements themselves hybridise. Due to long hybridisation domains ($\geq 28bp$), all reactions in the chemistry were considered irreversible at room temperature. Strand displacement reactions were approximated as elementary bi-molecular processes [6].

Supplementary Note 9.1.1 Rule Notation in Supplementary Table 3

Polymers are labelled in the direction shown in Supplementary Figure 14. Uppercase letters generally denote strands with secondary structure loops (e.g. *X* and *Y*), lower case letters denote strands without secondary structure. Symbol σ denotes a 'stack polymer' containing alternating *push* p and *write* w strands, and optionally beginning with a *start* s strand. Symbol σ is also allowed to represent an *empty polymer*. Brackets notation is used to indicate that the ends (or middle) of a stack polymer are specific strands. For example, $(w)\sigma(p)$ is any stack polymer beginning with w and ending with p. Possible polymers obeying this pattern are wp, $w\underline{pw}p$, $w\underline{pwpw}p$ and so on, where underlined strands represent the middle polymer section σ (in the first case, σ is empty). Conversely, $\sigma(p)$ is any stack polymer ending in p, such as p,$\underline{s}p$, $\underline{w}p$, $\underline{spw}p$, $\underline{pw}p$ and so on (again, in the first case, σ is empty). Subscript indexes i,j, k are used to identify physically distinct stack polymers, i.e. σ_i is a different molecule (with possibly different strand composition) to σ_j. A stack polymer written $\sigma_i(wp)\sigma_j$ is two different stack polymers joined together at some point in the middle with strands wp. When popping and reading, blunt-ended duplex waste products are formed. These are denoted $\vdash$duplex$\dashv$ and are considered non-reactive. Otherwise, the ends of stack complexes have "sticky" ssDNA overhangs and are reactive.

Species S denotes either a single *start* s strand or a *linker-start* complex ks. Species Z denotes either a signal X, a signal Y or a linear *write* w. The species substituted is consistent throughout the reaction, for example rule:

$$\sigma_i(Z) + (p)\sigma_j \xrightarrow{k_A} \sigma_i(Zp)\sigma_j$$

Folds out into three rules:

$$\sigma_i(X) + (p)\sigma_j \xrightarrow{k_A} \sigma_i(Xp)\sigma_j$$

$$\sigma_i(Y) + (p)\sigma_j \xrightarrow{k_A} \sigma_i(Yp)\sigma_j$$

$$\sigma_i(w) + (p)\sigma_j \xrightarrow{k_A} \sigma_i(wp)\sigma_j$$

Vertical bar notation also denotes "or": for example $(q|S)$ denotes either *pop* q or species S. Similarly, the species substituted is consistent throughout the reaction. For example rule:

$$\sigma((Z|S)p) + q \xrightarrow{k_2} \sigma(Z|S) + \vdash pq \dashv$$

Folds out into two rules:

$$\sigma(Zp) + q \xrightarrow{k_2} \sigma(Z) + \vdash pq \dashv$$

$$\sigma(Sp) + q \xrightarrow{k_2} \sigma(S) + \vdash pq \dashv$$

τ polymers (containing read r and pop q strands, and that optionally end with a start s strand) use the same notation as σ polymers. It should be noted that τ polymers that end with a *start* hybridised to a *linker* are written e.g. $qrqrks$, even though it is *start* and not *linker* that hybridises with the ultimate *read.* Finally, beads are not denoted in the reaction model since it is assumed that all *linkers* are attached to beads. Rules C1-C4 have special notation explained in the next section.

	Rule	Stack Op.	Description	
1	LH1	push	$S + (p)\sigma \xrightarrow{k_A} (Sp)\sigma$	σ stack hybridisation
2	LH2	push	$\sigma_i(Z) + (p)\sigma_j \xrightarrow{k_A} \sigma_i(Zp)\sigma_j$	
3	LH3	add signal	$\sigma_i(p) + (Z)\sigma_j \xrightarrow{k_{BC}} \sigma_i(pZ)\sigma_j$	
4	H1		$Z + r \xrightarrow{k_{ABC}} \vdash Zr \dashv$	Hybridisation to inert double helix
5	H2		$p + q \xrightarrow{k_{ABC}} \vdash pq \dashv$	
6	LH4		$\tau(r) + S \xrightarrow{k_A} \tau(rS)$	τ stack hybridisation
7	LH5		$\tau_i(r) + (q)\tau_j \xrightarrow{k_A} \tau_i(rq)\tau_j$	
8	LH6		$\tau_i(q) + (r)\tau_j \xrightarrow{k_{BC}} \tau_i(qr)\tau_j$	
9	SD1	read signal	$\sigma(pZ) + r \xrightarrow{k_1} \sigma(p) + \vdash Zr \dashv$	Right hand SD from σ stack
10	SD2	pop	$\sigma((Z\vert S)p) + q \xrightarrow{k_2} \sigma(Z\vert S) + \vdash pq \dashv$	
11	SD3		$r + (Zp)\sigma \xrightarrow{k_2} \vdash Zr \dashv + (p)\sigma$	Left hand SD from σ stack
12	SD4		$q + (pZ)\sigma \xrightarrow{k_1} \vdash pq \dashv + (Z)\sigma$	
13	SD5		$Z + (r(q\vert S))\tau \xrightarrow{k_2} \vdash Zr \dashv + (q\vert S)\tau$	Left hand SD from τ stack
14	SD6		$p + (qr)\tau \xrightarrow{k_1} \vdash pq \dashv + (r)\tau$	
15	SD7		$\tau(qr) + Z \xrightarrow{k_1} \tau(q) + \vdash Zr \dashv$	Right hand SD from τ stack
16	SD8		$\tau(rq) + p \xrightarrow{k_2} \tau(r) + \vdash pq \dashv$	
17	C1		$\sigma(pZ) + \tau(qr) \xrightarrow{k_A^{\text{chain}}} i \vdash Xr \dashv + j \vdash Yr \dashv + k \vdash wr \dashv + l \vdash pq \dashv + \omega$	Chain annihilation. (End hyb)
18	C2		$\sigma((Z\vert s)p) + \tau(rq) \xrightarrow{k_{BC}^{\text{chain}}} i \vdash Xr \dashv + j \vdash Yr \dashv + k \vdash wr \dashv + l \vdash pq \dashv + \omega$	(End hyb)
19	C3		$(Zp)\sigma + (r(q\vert s))\tau + \xrightarrow{k_{BC}^{\text{chain}}} i \vdash Xr \dashv + j \vdash Yr \dashv + k \vdash wr \dashv + l \vdash pq \dashv + \omega$	(Beginning hyb)
20	C4		$(pZ)\sigma + (qr)\tau \xrightarrow{k_A^{\text{chain}}} i \vdash Xr \dashv + j \vdash Yr \dashv + k \vdash wr \dashv + l \vdash pq \dashv + \omega$	(Beginning hyb)

Supplementary Table 3: Complete set of 20 reaction rules considered for the DNA stack chemistry with washing. See Supplementary Note 9.1.1 for notation. Column “Stack Op.” shows which stack operation is associated with the rule (if any). All rules entail bi-molecular reactions. In the Gillespie algorithm, the stochastic rate constant for bi-molecular reactions with different reactants is set to $c = k/N_AV$, where k is the corresponding kinetic rate constant and N_A is Avogadro’s Number. Where bi-molecular reactions had identical reactants (a possibility for reaction rules LH2, LH3, LH5, LH6), $c = 2k/N_AV$. Rate setting was handled automatically by `stocal` (https://github.com/harfel/stocal). *Linker-start* and *linker-releaser* hybridisation rules are not included as these reactions were not explicitly simulated.

Supplementary Note 9.1.2 Note on Chain Annihilation of σ and τ Polymers

For completeness, rules C1-C4 were included in the model to represent that case when σ and τ stacks reacted with *each other*, as opposed to just reacting with single monomers. These "chain annihilation" events are a complex multi-stage reaction that we hypothesised to occur when a σ and τ stack hybridise at their "start strand" end (black dots in figure) or at the opposite end to their "start strand" end. After the initial hybridisation event, a chain of four-way strand displacement reactions is initiated by fraying ends at high effective concentration relative to each other. The fraying ends first hybridise and then cause four-way branch migration. This process is repeated over and over again producing $\vdash Zr \dashv$ and $\vdash pq \dashv$ duplexes alternately until either one or both polymers have no more strands. The irreversible production of blunt end duplexes drives the reaction forwards.

The notation (e.g. rule C1)

$$\sigma(pZ) + \tau(qr) \overset{k_A^{\text{chain}}}{\to} i \vdash Xr \dashv + j \vdash Yr \dashv + k \vdash wr \dashv + l \vdash pq \dashv + \omega$$

means that a σ stack ending with a signal hybridises with a τ polymer ending with a *read* to eventually form $i\times \vdash Xr \dashv$, $j\times \vdash Yr \dashv$, $k\times \vdash wr \dashv$ and $l\times \vdash pq \dashv$ non-reactive products and also a residual polymer ω. Stoichiometric coefficients i, j, k, l may be 0 and the residual polymer ω may be empty: they depend on the exact composition of the reacting σ and τ, and were determined algorithmically as follows. For rules C1 and C2, both reactants are aligned at their right hand ends (because the initial hybridisation event is at the "end" side of each complex, Supplementary Figure 14), and all pq and Zr pairs (blue) are designated as products. The residual polymer on the left hand side (black) is designated as ω.

Rule C1 example reaction (hybridisation on A domain at end of both complexes).

$$spw + qrqr \overset{k_A^{\text{chain}}}{\to} \vdash wr \dashv + \vdash pq \dashv + qrs \quad (i = 0, j = 0, k = 1, l = 1, \omega = qrs)$$

σ		s	p	w
		|	|	|
τ	q	r	q	r

Rule C2 example reaction (hybridisation on BC domain at end of both complexes).

$$XpYp + rqrq \overset{k_{BC}^{\text{chain}}}{\to} \vdash Xr \dashv + \vdash Yr \dashv + 2 \vdash pq \dashv \quad (i = 1, j = 1, k = 0, l = 2, \omega = \text{none})$$

σ	X	p	Y	p
	|	|	|	|
τ	r	q	r	q

For rules C3 and C4, both reactants are aligned at their left hand ends (because the initial hybridisation event is at the "beginning" side of each complex, Supplementary Figure 14), and all pq and Zr pairs (blue) are designated as products. The residual polymer on the right hand side (black) is designated as ω.

Rule C3 example reaction (hybridisation on BC domain at beginning of both complexes).

$$YpXpXp + rqrs \overset{k_{BC}^{\text{chain}}}{\to} \vdash Xr \dashv + \vdash Yr \dashv + \vdash pq \dashv + spXp \quad (i = 1, j = 1, k = 0, l = 1, \omega = spXp)$$

σ	Y	p	X	p	X	p
	|	|	|	|		
τ	r	q	r	s		

Rule C4 example reaction (hybridisation on A domain at beginning of both complexes).

$$pYpY + qr \xrightarrow{k_A^{\text{chain}}} \vdash Yr \dashv + \vdash pq \dashv + pY \quad (i=0, j=1, k=0, l=1, \omega = pY)$$

σ	p	Y	p	Y
	|	|		
τ	q	r		

A chain annihilation reaction was approximated as a single step bimolecular reaction whose rate constant was set to the rate constant of the initial hybridisation event as an upper bound i.e. $k_A^{\text{chain}} = k_A$, $k_{BC}^{\text{chain}} = k_{BC}$. However, even at upper bound values, simulation runs suggested that these chain annihilation events were relatively rare - at least for our sepharose bead washing parameters $\mu = 0.1$, $\phi_0 = 0.33$ (Supplementary Note 9.4).

A further complex case not considered is when σ and τ polymers hybridise at two ends simultaneously.

Supplementary Note 9.1.3 Reactions Not Included

- Stack circularisation reactions were not included, e.g. $XpXpXpXp \rightarrow \text{nicked dsDNA loop}$.
- Zero-toehold strand displacement reactions initiated by strand fraying (leak reactions) were not included. Hence, stacks with ssDNA dangling ends were not able to invade existing stacks (or themselves) through this mechanism. We note that including the zero-toehold reaction type greatly increases the possible reactions in the chemistry.
- Generally, no reactions between stack polymers (except for chain annihilation) were included.

Additionally, the model assumed no synthesis errors in the strands or extra synthesis byproducts.

Supplementary Note 9.2 Parameter Estimation

Kinetics of the model are governed by five molecular and two operational parameters:

1. k_A, the bi-molecular hybridisation rate constant of the 28bp *A* domain to its complement.
2. k_{BC}, the bi-molecular hybridisation rate constant of the 28bp *BC* domain to its complement.
3. k_{ABC}, the bi-molecular hybridisation rate constant of the 56bp *ABC* domain to its complement.
4. k_1, the bi-molecular strand displacement rate constant when the *A* domain acts as toehold.
5. k_2, the bi-molecular strand displacement rate constant when the *BC* domain acts as toehold.

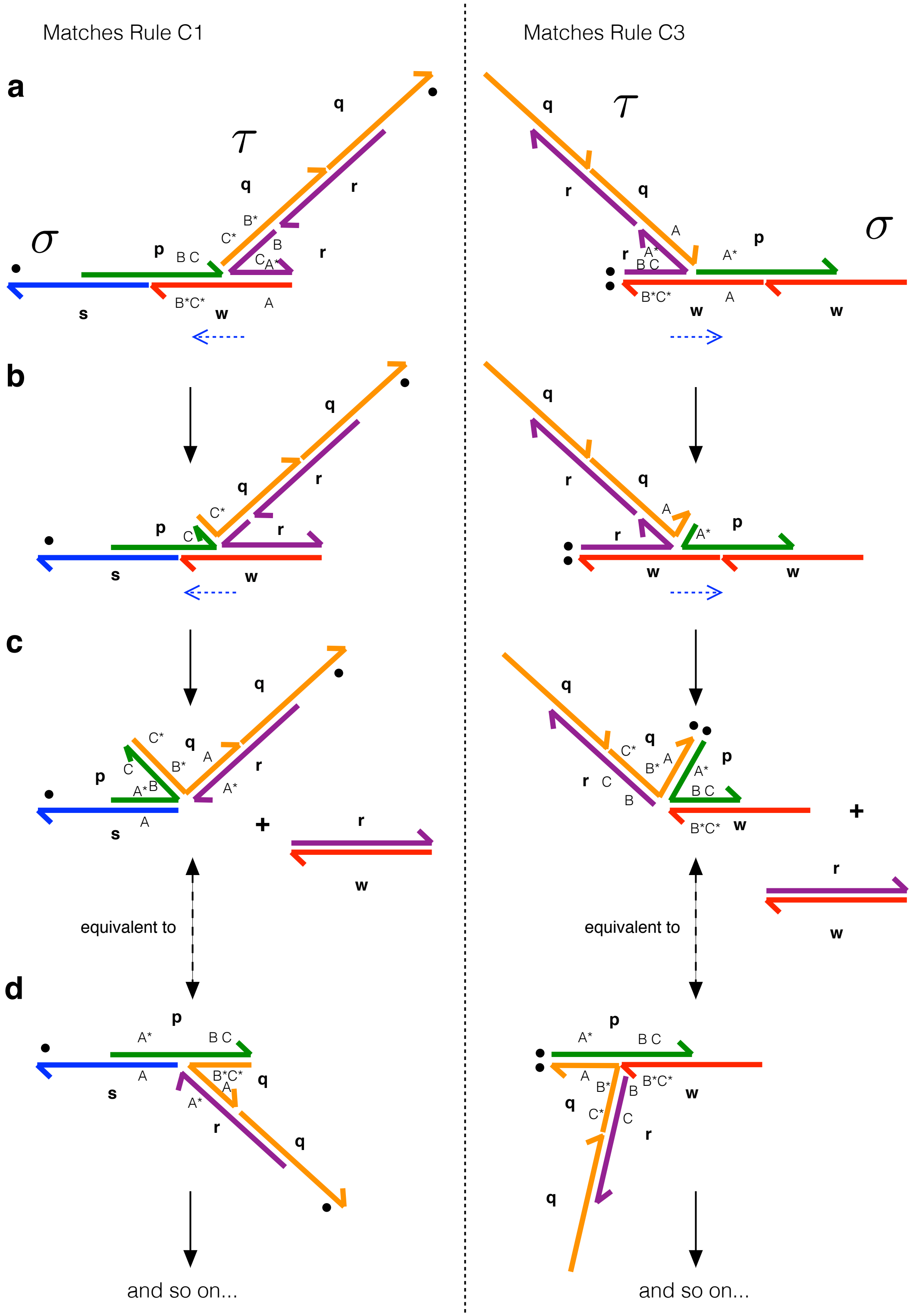

Supplementary Figure 15: Examples of a hypothesised "chain annihilation" multi-stage reaction when a σ stack hybridises with a τ polymer. Reaction proceeds (a) to (c), with (d) showing (c) in a different but equivalent orientation. To help with orientation, black dots at the ends of polymers identify the end where *start* is or would be added. Blue dotted arrows indicate direction of four-way branch migration. Here, σ stacks feature only linear *write* signals, but could also feature looped X or Y signals in their place.

6. $0 \leq \mu \leq 1$, the fraction sepharose bead mass lost on each washing cycle. 0 = no beads lost, 1 = all beads lost.

7. $0 \leq \phi_0 \leq 1$, the initial volume fraction of supernatant DNA species surviving the washing cycle via non-specific bead binding. 0 = no species in supernatant bind non-specifically to beads, 1 = all species in supernatant bind non-specifically to beads.

Supplementary Note 9.2.1 Hybridisation and Strand Displacement Rate Constants

Bi-molecular rate constants k_A, k_{BC} and k_{ABC} were determined via UV absorption experiments (Supplementary Note 8). Bi-molecular strand displacement rate constants k_1 and k_2 were assumed equal to hybridisation constants k_A and k_{BC} respectively, due to the long 28nt "toeholds" of the strand displacement reaction. The latter assumption was motivated by the fact that the effective bi-molecular rate of a strand displacement reaction saturates at the forward hybridisation rate of the toehold, when the toehold is long and binds irreversibly [6].

Kinetic rate constants were considered to be independent of the length of polymer complexes involved in a reaction (i.e differential diffusion - or variation in diffusion - and steric hindrance were assumed negligible).

Supplementary Note 9.2.2 Washing Parameters μ and ϕ_0

Washing parameters μ and ϕ_0 were the two free parameters of the model that were collectively fitted from experimental gel electrophoresis data.

Parameter μ is the constant fraction of beads lost on each wash. Parameter ϕ_0 is the initial *volume fraction* of the supernatant solution transferred through the wash cycle to the next reaction stage (when all beads still exist). Species in this volume fraction are assumed to be non-specifically attached to the beads. As less beads exist, less volume fraction of the supernatant is transferred. At washing n, parameter ϕ_0 is multiplied by the remaining (normalised) bead mass B_n to give the effective volume fraction transferred:

$$\phi_n = \phi_0 B_n \tag{8}$$

where $B_n = (1 - \mu)^n$. The bead mass that all reactions start with before any washing corresponds to a normalised bead mass of $B_0 = 1.0$. The supernatant is considered well-stirred such that transferring fraction ϕ of the supernatant volume is equivalent to saying that fraction ϕ of *each individual supernatant species* is transferred. Bead loss and species transferral are modelled as deterministic processes.

It is worth noting that this approach does not consider how the concentration of an individual species could affect its partitioning to beads. All species partition with the same fraction regardless of concentration. A more accurate approach could be to consider an equilibrated dissociation $s_i.b \overset{K_d}{\rightleftharpoons} s_i + b$ existing between each species s_i and the beads b, and to use dissociation constant K_d (in nanomolar units) as the free model parameter instead of ϕ_0. In this way, partitioning would be concentration dependent for each species, and there would be a natural shift in partitioning as beads are lost. However, this latter approach would assume that the strand-bead equilibrium is established before the end of the reaction, which may not be true for short wait times. Also K_d is more difficult to interpret as a measure of how many species are non-specifically transferred through the wash. Therefore, the simpler “volume fraction” approach was adopted.

Washing parameters μ and ϕ_0 were set by comparing model output (averaged over n=10 runs) to 17 washing experiments on stacks using the linear *write* strand (polyacrylamide gels in Figure 2a, b, c of

main paper). For a parameter set (μ, ϕ_0), the final model prediction for an experiment was converted to a gel banding pattern by leveraging the linear electrophoretic mobility behaviour of linear stacks (Supplementary Figure 13). The model banding pattern was then converted to a qualitative "intensity ordering" inequality that classified bands in descending order of their mass concentration of DNA. The corresponding experimental banding pattern was similarly converted to an intensity ordering inequality (assuming SYBR Gold intercalates into ssDNA and dsDNA equally). For each of the 17 experiments, a (μ, ϕ_0) pair was found that minimised the least squares error between the model intensity ordering and the experimental gel intensity ordering inequalities. Each experiment suggested differing optimal (μ, ϕ_0). Final parameters were obtained as the (μ, ϕ_0) pair with the lowest average normalised error over all 17 experiments, and our experimental operation point was found to be $(\mu, \phi_0) = (0.1, 0.33)$ (although a broader region of other possible combinations existed). Importantly, this fitting method did not require estimation of the *absolute* mass concentrations of bands on polyacrylamide gel.

For the 17 experiment fitting, the lowest error parameter point (μ, ϕ_0) was found to be sensitive to rate constant k_{BC}. For example, increasing k_{BC} from $3 \times 10^4 M^{-1}s^{-1}$ to $1 \times 10^6 M^{-1}s^{-1}$ caused $(\mu, \phi_0) = (0.05, 0.25)$. For different k_{BC}, increasing k_A had no effect on (μ, ϕ_0).

Supplementary Note 9.3 Simulation

Nanomolar concentrations used in experiments constrained the simulation volume to be set to $V = 1.5 \times 10^{-13}$ litres (0.15 picolitres), in order to obtain particle numbers that on the one hand avoided small system size artefacts, and on the other had feasible computation times in python (300nM $\approx$ 27100 particles). This volume represents the *total* solution volume, i.e. volume of supernatant solution + volume of sepharose bead solution.

Supplementary Note 9.3.1 Initial Condition

The initial condition of the stochastic simulation is set to the assumed state of the DNA chemistry *after* the following steps had been performed: (i) *linker* incubated with sepharose beads for sufficient time to allow all *linker* strands to bind to beads, (ii) wash, (iii) *start* added and reaction stirred for sufficient time to allow all *start* strands to hybridise with *linker*, (iv) wash.

1. In step (i), *linker* strands are added at an initial particle number

$$n_{k_0} = [k]_0 N_A V \tag{9}$$

   where $[k]_0$ represents the initial *linker* concentration. During incubation with beads, **all** biotinylated *linker* strands are considered to irreversibly attach to the streptavidin binding sites on the beads. For sepharose beads with a binding capacity of 300nmol biotin per ml solution, $200ul$ reaction solutions made up of $100ul$ of beads would contain $\approx 150\mu M$ effective concentration of streptavidin binding sites: i.e. far in excess of *linker* strands at $200nM$. In the presence of so many specific binding sites, non-specific binding of the *linker* to the beads was assumed not to take place.

2. In wash step (ii), the *linker* particle number is multiplied by $1 - \mu$, to model losing μ percent of the sepharose beads.

3. In reaction step (iii), *start* is added at the following initial particle number:

$$n_{s_0} = [s]_0 N_A V \tag{10}$$

where $[s]_0$ represents the initial *start* concentration. It is assumed that the irreversible *linker-start* reaction proceeded to 100% completion over the wait time. The remaining excess of *start* particles at the end of the reaction is calculated as:

$$n_{s_{\text{surplus}}} = n_{s_0} - (1-\mu)n_{k_0} \tag{11}$$

where a negative surplus represents the converse case of an excess of *linker* particles.

4. In wash step (iv), the particle number of all bead-bound species (*linker* and *linker-start*) is again multiplied by $1-\mu$ to model bead loss. Also, $\phi_0 \times B_2$ volume fraction of the excess of *start* particles $n_{s_{\text{surplus}}}$ are considered to survive the wash by non-specific binding to the sepharose beads.

Considering steps (i)-(iv), the following formulas for the initial conditions for the simulation are arrived at:

- If *start* is in excess of, or equal to, the bound *linker*, i.e. if $n_{s_{surplus}} \geq 0$, then the initial condition for simulation is:

$$n_k = 0 \qquad n_{ks} = (1-\mu)^2 n_{k_0} \qquad n_s = \phi_0 B_2 n_{s_{\text{surplus}}} \tag{12}$$

  where n_k, n_{ks}, n_s are the particle numbers of bead-bound *linker*, bead-bound *linker-start* and free *start*, respectively, at the beginning of the simulation.

- On the other hand, if the bound *linker* is in excess of *start*, i.e. if $n_{s_{\text{surplus}}} < 0$, then the initial condition for simulation is:

$$n_k = -(1-\mu)n_{s_{\text{surplus}}} \qquad n_{ks} = (1-\mu)n_{s_0} \qquad n_s = 0 \tag{13}$$

In both cases, the normalised bead mass remaining before the simulation begins is $B_2 = (1-\mu)^2$.

Supplementary Note 9.3.2 Simulation Algorithm

1. Set initial condition particles numbers as described above, depending on whether *start* is in excess of *linker* or vice versa.
2. Add first strand b after *start* at particle number $n_b = [b]_0 N_A V$
3. Simulate 30 minutes of reaction time (Gillespie Direct SSA)
4. If supernatant state required, report it
5. **Wash**
6. Add next strand b at particle number $n_b = [b]_0 N_A V$
7. Simulate 30 minutes of reaction time (Gillespie Direct SSA)
8. If supernatant state required, report it
9. If there are more strands to add, go to Step 5: but, if next strand is *releaser*, continue
10. **Wash**

11. Simulate final 30 minutes of reaction time (Gillespie Direct SSA)
12. Instantaneously make the *linker-releaser* reaction go to 100% completion: all species bound to beads go into supernatant (this assumes releaser is in excess). Add any surplus *releaser* to supernatant. The *linker-releaser* double stranded complexes stay anchored to the beads and do not enter the supernatant.
13. Report supernatant state and stop.

During **Wash**, the following happens:

- Bead loss: All species bound to beads specifically, i.e. species beginning with *linker*, have their particle number decreased to $(1-\mu)n_x$, where n_x is the particle number of a bead-bound stack species x.
- Bead loss: The normalised mass of sepharose beads remaining is set to $B_n = (1-\mu)^n$ where n is the number of the current wash.
- Non-specific bead binding: The volume fraction of supernatant transferred is set to $\phi_n = \phi_0 B_n$. Species not bound to beads (i.e. species not beginning with *linker*) have their particle number set to $\phi_n n_y$, where n_y is the particle number of a non bead-bound species y.

Simulation step 11 does not include the strand displacement of bead-bound stacks by *linker* monomers, and so the concentration kinetics of some species at this step are not accurate. However, in this model, bead-bound species beginning *linker-start* have the same reactivity as free-floating species beginning *start*, and so it follows that after step 12, the correct final state of the system is still arrived at. Step 12 assumes that *releaser* is in excess to release all bead-bound stacks.

Supplementary Note 9.3.3 Simulation Dry Run Example

A dry run of the simulation algorithm for reaction sequence k - ● - s - ● - p - ● - x - ● - z - ○ is given below for clarity, where $[k] = [z] = 200nM$, $[s] = [p] = [x] = 300nM$ and $\mu = 0.1,\ \phi_0 = 0.33$.

- For **step 1** of simulation algorithm, the initial condition for the reaction is calculated as follows:
  - $200nM$ of *linker* binds specifically to beads during the wait time. This corresponds to $n_{k0} = 200 \times 10^{-9} \times N_A \times V = 18066$ *linker* strands bound to beads in the simulation.
  - Wash 1: The supernatant solution is poured out and 10% of beads are lost ($\mu = 0.1$). The normalised mass of beads remaining is $B_1 = (1-0.1) = 0.9$ after the wash. The number of *linker* strands bound to the remaining beads (now out of solution) is $n_k = 18066 \times 0.90 = 16259$.
  - *Start* is added at $300nM$, refilling the solution volume to V. This corresponds to $n_{s0} = 300 \times 10^{-9} \times N_A \times V = 27099$ *start* strands added to the reaction.
  - Over 30 minutes reaction time, *start* hybridises with all *linker* complexes bound to beads, giving $n_{ks} = 16259$ *linker-start* complexes at the end of the reaction. The excess number of *start* single strands remaining after the reaction is $n_{s_{\text{surplus}}} = 27099 - 16259 = 10840$. The number of *linker* single strands remaining is $n_k = 0$ (they are all complexed with start).
  - Wash 2: The supernatant solution is poured out again and 10% of beads are lost. The number of *linker-start* strands surviving on the remaining beads is $n_{ks} = 16259 \times (1-0.1) = 14633$.

- The normalised bead mass after the second wash is $B_2 = 0.90 \times (1 - 0.1) = 0.81$. When $\phi_0 = 0.33$ this means that $\phi_2 = 0.33 \times 0.81 = 0.2673$, i.e. approximately 27% of each supernatant species will non-specifically bind to beads and be transferred through the wash to the next reaction stage. Therefore $n_s = 10840 \times 0.2673 = 2897$ *start* strands will be transferred to the next reaction stage.
- Verifying formulas (12), the initial condition after the second wash is:

$$n_k = 0$$

$$n_{ks} = (1 - \mu)^2 n_{k_0} = (1 - 0.1)^2 \times 18066 = 14633$$

$$n_s = \phi_0 B_2 n_{s_{\text{surplus}}} = 0.33 \times (1 - 0.1)^2 \times 10841 = 2897$$

- Reaction sequence k - ● - s - ● has so far been completed.

- In **step 2**, *push* is added as $n_{p0} = 300 \times 10^{-9} \times N_A \times V = 27099$ strands, refilling the solution volume to V.
- In **step 3**, the reaction is simulated stochastically for 30 minutes. For the rate constants in Supplementary Note 9.2.1, a typical run gives 2897 *push* strands hybridising with free *start* strands, 14621 *push* strands hybridising with *linker-start* complexes attached to the beads and 9581 *push* strands not hybridising, yielding final state:

$$n_{ks} = 12$$

$$n_{ksp} = 14621$$

$$n_p = 9581$$

$$n_{sp} = 2897$$

- **Step 4** is skipped.
- In **step 5** the system is washed.
  - Wash 3: 10% of the beads are lost. Hence, 10% of all species starting *linker* are removed from the system. After the wash, their copy numbers will be:

$$n_{ks} = 12 \times (1 - 0.1) = 10$$

$$n_{ksp} = 14621 \times (1 - 0.1) = 13158$$

– The normalise mass of beads after the third wash is $B_3 = 0.81 \times (1 - 0.1) = 0.729$. Hence $\phi_3 = 0.33 \times 0.729 = 0.24057$, i.e. approximately 24% of each supernatant species will be transferred through the wash by non-specific binding to beads. After the wash, their copy numbers will be:

$$n_p = 9581 \times 0.24057 = 2304$$

$$n_{sp} = 2897 \times 0.24057 = 696$$

– Reaction sequence k-●-s-●-p-● has so far been completed.

- In **step 6**, *X* is added as $n_{X0} = 300 \times 10^{-9} \times N_A \times V = 27099$ strands, refilling the solution volume to V.
- In **step** 7, the reaction is simulated stochastically for 30 minutes. In this case, the *push* strands carried through the previous wash by non-specific attachment to the beads begin to cause polymerisation reactions with *X*. As a result, the system state at the end of the reaction is more complex, with a typical example being:

$$n_{ks} = 10$$

$$n_{ksp} = 10$$

$$n_X = 10003$$

$$n_{kspX} = 12049$$

$$n_{kspXpX} = 1011$$

$$n_{kspXpXpX} = 79$$

$$n_{kspXpXpXpX} = 9$$

$$n_{spX} = 634$$

$$n_{spXpX} = 59$$

$$n_{spXpXpX} = 3$$

$$n_{Xp} = 1$$

$$n_{XpX} = 864$$

$$n_{XpXpX} = 74$$

$$n_{XpXpXpX} = 10$$

- **Step 8** is skipped.
- In **step 9** the next strand is *releaser*, so step 10 is executed next.
- In **step 10** the system is washed.
  - Wash 4: 10% of the beads are lost. Hence, 10% of all species starting *linker* are removed from the system. After the wash, their copy numbers will be:

$$n_{ks} = 10 \times (1 - 0.1) = 9$$

$$n_{ksp} = 10 \times (1 - 0.1) = 9$$

$$n_{kspX} = 12049 \times (1 - 0.1) = 10844$$

$$n_{kspXpX} = 1011 \times (1 - 0.1) = 909$$

$$n_{kspXpXpX} = 79 \times (1 - 0.1) = 71$$

$$n_{kspXpXpXpX} = 9 \times (1 - 0.1) = 8$$

  - The normalise mass of beads after the fourth wash is $B_4 = 0.729 \times (1 - 0.1) = 0.6561$. Hence $\phi_4 = 0.33 \times 0.6561 = 0.21651$ approximately 22% of each supernatant species will be transferred through the wash by non-specific binding to beads. After the wash, their copy numbers will be:

$$n_X = 10003 \times 0.21651 = 2165$$

$$n_{spX} = 634 \times 0.21651 = 137$$

$$n_{spXpX} = 59 \times 0.21651 = 12$$

$$n_{spXpXpX} = 3 \times 0.21651 = 0 \text{ (eliminated)}$$

$$n_{Xp} = 1 \times 0.21651 = 0 \text{ (eliminated)}$$

$$n_{XpX} = 864 \times 0.21651 = 187$$

$$n_{XpXpX} = 74 \times 0.21651 = 16$$

$$n_{XpXpXpX} = 10 \times 0.21651 = 2$$

– Reaction sequence k - ● - s - ● - p - ● - X - ● has so far been completed.

- In **step 11**, the system is simulated stochastically for a further 30 minutes, without the addition of the *releaser* strand.
  – Reaction sequence k - ● - s - ● - p - ● - X - ● - ○ has so far been completed.
- In **step 12**, the *releaser* reaction is then applied instantaneously. *Releaser* is added at strand copy number $n_{z0} = 200 \times 10^{-9} \times N_A \times V = 18066$. The *releaser* is assumed in excess of all surviving bead-bound complexes in the system, and therefore releases them all.
  – All stack complexes bound to beads go into supernatant solution.
  – Any excess *releaser* strands not used stay in supernatant solution.
  – *Linker-releaser* complexes stay anchored to beads and don't go into supernatant.
  – This yields final supernatant state:

$$n_s = 9$$

$$n_{sp} = 2$$

$$n_X = 2158$$

$$n_{spX} = 10988$$

$$n_{spXpX} = 921$$

$$n_{spXpXpX} = 71$$

$$n_{spXpXpXpX} = 8$$

$$n_{XpX} = 187$$

$$n_{XpXpX} = 16$$

$$n_{XpXpXpX} = 2$$

$$n_z = 6216$$

  – Reaction sequence k - ● - s - ● - p - ● - X - ● - ○- z (instantaneous) has so far been completed which, when *releaser* is in excess, has the same end state as target sequence k - ● - s - ● - p - ● - X - ● - z - ○.
- The final supernatant state is reported in **step 13**.

Supplementary Note 9.4 Analysis of Rule Firing Counts at $\mu = 0.1,\ \phi_0 = 0.33$

Average rule fire counts (n=50 repeats) in simulations of operations sequences *seq1*, *5*, *10*, *20* and *seqR* for our experimental washing efficiency ($\mu = 0.1,\ \phi_0 = 0.33$) are shown in Supplementary Figure 16 and Supplementary Table 4. It can be concluded:

- All rules in the full 20-rule model are fired at some time, non are redundant.
- LH1, LH2, LH3 are the most firing rules in all cases.
- The operations sequences *seq1, 5, 10, 20* and *seqR* could be captured well by a reduced model (11 rules: LH1,2,3; H1,2; LH5,6; SD1,2,5,6).
- All operations sequences *except seq1* could also be captured well by a further reduced model (7 rules: LH1,2,3; LH5,6; SD1,2).
- A five rule minimal model is too simple to capture interactions in the chemistry.
- Chain annihilation rules C1-C4 fire very rarely, accounting for <1% of fires in all cases.
- Strand displacement reactions at the "wrong end" of stack polymers are negligible, accounting for <1% of fires in all cases (wrong end = left hand end of σ stacks, right hand end of τ stacks).
- Reactions involving σ stacks are dominant over reactions involving τ stacks in all cases. (This is expected by the way the system is operated)
- Rules for τ stacks are mostly required when a small number of signals are recorded before popping begins.

As $\phi_0 \to 1$ (effectively no washing) there will be more species in the reaction and the minority rules are expected to fire more.

Rule Group	Rules	% Percentage of total rule fires				
		seq1	seq5	seq10	seq20	seqR
Hybridisation of σ stacks	LH1,2,3	46.24	70.75	88.71	98.95	70.2
SD at right hand end of σstack	SD1,2	27.43	15.63	5.00	0.17	16.80
SD at left hand end of σ stack	SD3,4	0.27	0.05	0.01	0	0.13
Hybridisation of τ stacks	LH4,5,6	12.35	11.43	5.81	0.83	8.58
SD at left hand end of τ stack	SD5,6	3.87	0.35	0.01	0	0.14
SD at right hand end of τ stack	SD7,8	0.24	0.01	0	0	0.09
DNA duplex hybridisation	H1,2	8.99	1.27	0.40	0.05	3.55
Chain Annihilation	C1,2,3,4	0.61	0.51	0.06	0	0.50
All rules involving σ stacks		74.55	86.94	93.78	99.12	87.63
All rules involving τ stacks		17.07	12.29	5.88	0.83	9.31
Reduced Model	LH1,2,3; H1,2; LH5,6; SD1,2,5,6	97.19	99.25	99.92	100	99.26
Further Reduced Model	LH1,2,3; LH5,6; SD1,2	84.32	97.63	99.52	99.95	95.56
Minimal Model	LH1,2,3; SD1,2	73.67	86.38	93.71	99.12	87.01

Supplementary Table 4: Firing rate of reaction rule *groups*, for each of the five operation sequences. Colour code: **First**, **Second**, **Third** most fired rule group in each column.

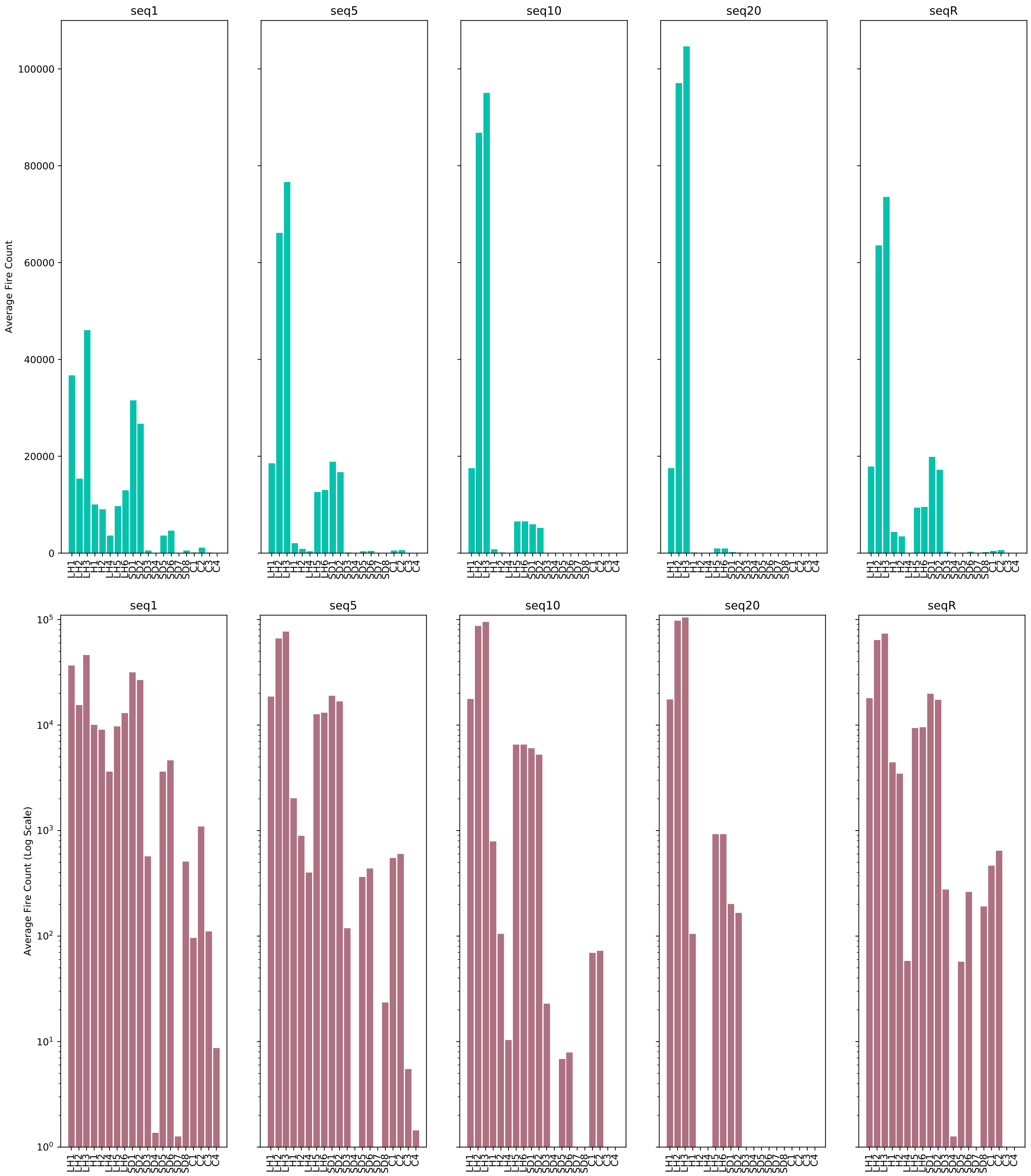

Supplementary Figure 16: Average count of reaction rule fires during simulation of operation sequences *seq1*, *5*, *10*, *20* and *seqR*. Washing parameters are $\mu = 0.1,\ \phi = 0.33$. Top: normal scale to emphasise most firing rules. Bottom: log scale to show all invoked rules.

Supplementary Note 9.5 Useful Calculations on Model Washing Procedure

Supplementary Note 9.5.1 Bead Half Life

Starting from any arbitrary normalised bead mass $0 \leq A_0 \leq 1$, after after n washes the normalised bead mass is:

$$A_n = A_0(1-\mu)^n$$

In this exponential decay, the number of washes $n_{1/2}$ at which the original normalised bead mass halves is given by:

$$A_n = A_{n_{1/2}} = \frac{1}{2}A_0$$

Solving

$$A_0(1-\mu)^{n_{1/2}} = \frac{1}{2}A_0$$

for $n_{1/2}$ gives:

$$n_{1/2} = log_{(1-\mu)}\left(\frac{1}{2}\right)$$

Using the logarithm base change rule $log_b(x) = log_c(x)/log_c(b)$, the above can be written:

$$n_{1/2} = \frac{ln\,(1/2)}{ln(1-\mu)} \tag{14}$$

For example when $\mu = 0.1$, $n_{1/2} = 6.6$ washes.

Supplementary Note 9.5.2 Supernatant Species Survival

An estimate of how many washes m an arbitrary species S in the supernatant solution will survive for (simply being carried through washes by non-specific binding to the beads) can be derived as follows. If species S is introduced when n washes have already been completed (affecting the number of beads remaining), and S is assumed not to react with other species (either tethered to beads or in the supernatant), then:

- Before the first wash is performed ($m = 0$) there are S_0 particles of S in supernatant, calculated as $S_0 = N_A V[s]_0$ where V is the simulation volume and $[s]_0$ is the concentration of S.
- After $m = 1$ wash, there are $S_1 = \phi_1 S_0$ particles of S, where $\phi_1 = B_n(1-\mu)\phi_0$ is the non-specific transferral fraction and where $B_n = (1-\mu)^n$ is the normalised bead mass remaining at the time S is introduced.
- After $m = 2$ washes, there are $S_2 = \phi_2 S_1$ particles of S, where $\phi_2 = B_n(1-\mu)^2\phi_0$
- After $m = 3$ washes, there are $S_3 = \phi_3 S_2$ particles of S, where $\phi_3 = B_n(1-\mu)^3\phi_0$
- and so on...

It can be observed that the transferral fraction ϕ is not constant but rather decays exponentially as the number of washes advances. Hence, even at high concentration excess (e.g. $10\mu M$), introduced species will tend to get washed out of the supernatant only 1 or 2 washes later than will species at low concentration excess (e.g. $100nM$).

In general, the number of particles of S remaining after m washes is:

$$S_m = B_n^m (1-\mu)^{\frac{m(m+1)}{2}} \phi_0^m S_0 \tag{15}$$

The (approximate) number of washes m before species S completely disappears is found solving the above equation numerically: by incrementing m and stopping when $S_m < 1$. Supplementary Figure 17 performs this latter calculation to show an estimation of how quickly a supernatant species S gets washed out of the system under various conditions.

In practice, a strand introduced into the DNA stack chemistry will also react with stack species tethered to beads and with other species in supernatant (where sometimes multiple copies of the introduced strand will be included in a single polymer complex), and so the situation is more nuanced. However, the above calculation (15) gives a first approximation of the number of washes needed to flush a strand from supernatant, after it is introduced.

Note that m calculated from equation (15) depends on the simulation volume V through the initial particle number $S_0 = N_A V[s]_0$. Enlarging the simulation volume beyond $V = 1.5 \times 10^{-13}$ litres (0.15 picolitres) indeed increases the absolute number of washes m required to entirely flush species S from supernatant. However, the extra washes required only contain vanishingly small concentrations of S. Also, regardless of the volume used, the relative difference in the total number of washes required to flush S from supernatant when it is introduced at different concentrations $[s]_0$ remains the same.

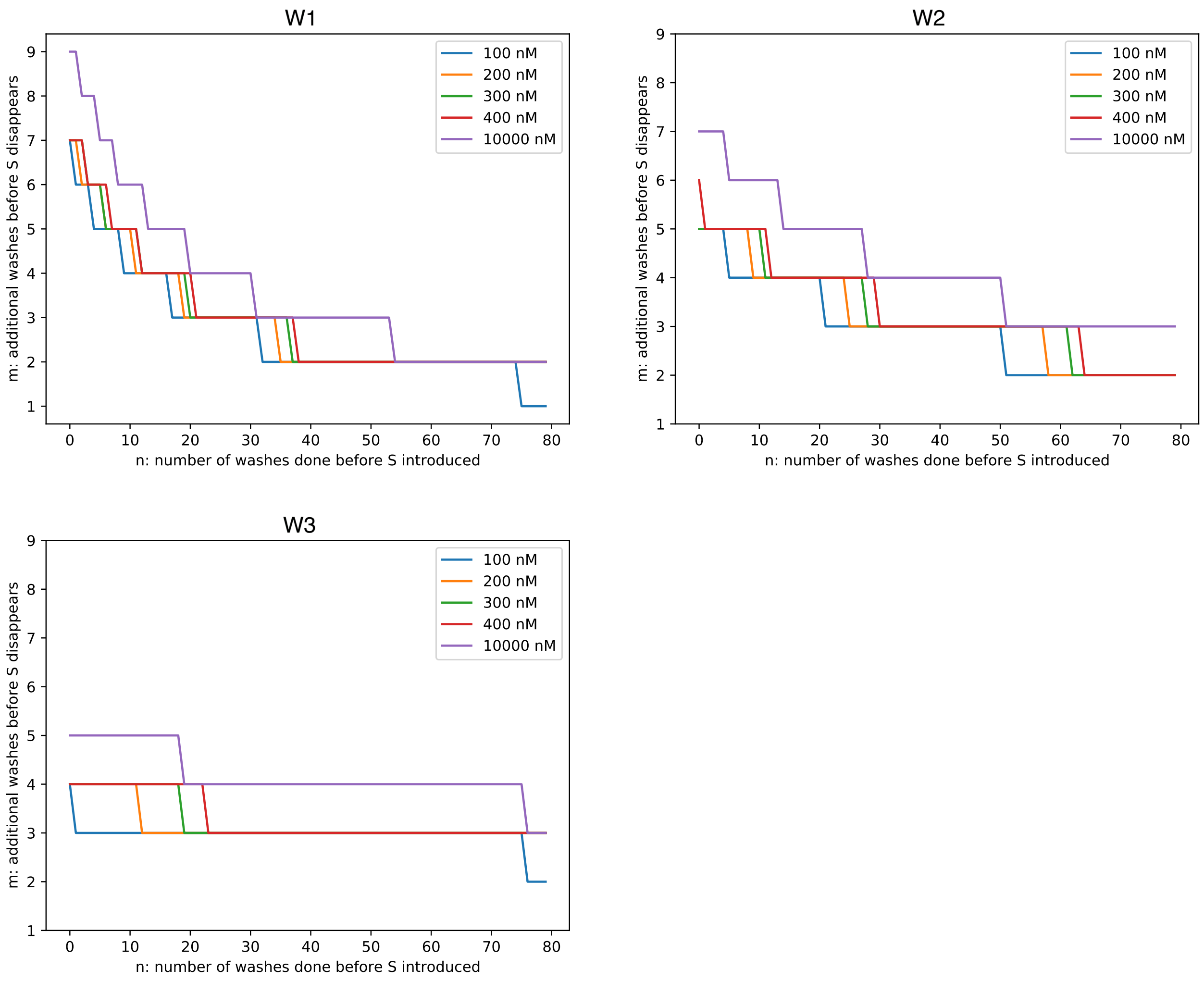

Supplementary Figure 17: Estimations of supernatant survival (for simulation volume $V = 1.5 \times 10^{-13}$ litres). The number of washes m that an arbitrary species S survives in supernatant (is carried through each wash by non-specific bead binding), given that S is added after n washes have already been completed. Separate graphs show different washing efficiencies. Coloured lines represent different concentrations $[s]_0$ at which species S is introduced.

Supplementary Note 10 Expanded Model Results

Supplementary Note 10.1 Most Robust Stack Read-out is Majority Signal Popped into Supernatant

This section demonstrates that the most robust read-out from the DNA stack, as predicted by the model, is the majority signal popped into supernatant (*Xr* or *Yr*) following the addition of *read* strands. First, 6 metrics for the stack (including performance metrics) are defined. Supplementary Note 10.1.2 then shows how these metrics change step-by-step as the DNA stack executes *seq1*, *seq5*, *seq10*, *seq20* and *seqR* under washing regimes W1, W2 and W3. Results are discussed in Supplementary Note 10.1.3.

Supplementary Note 10.1.1 Performance Metrics

Each operations sequence has the following 6 metrics calculated on the model state following each operation. See Supplementary Figure 18 for how an operations sequence corresponds to an underlying reaction sequence and the exact points at which the metrics are calculated. Below, "target stack" species σ_t refers to the sole stack species expected in solution after an operation is complete, in the case of *ideal operation* of the device.

1. **Purity** is the fraction of the total DNA strands in the system which constitute the target stack σ_t . Defined as:

$$\mathcal{P} = \frac{\sigma_{ss}}{n_{ss}} \qquad 0 \leq \mathcal{P} \leq 1 \tag{16}$$

   where σ_{ss} and n_{ss} are the number of single strands existing in the target stack species σ_t and in the total system, respectively. Note that n_{ss} omits strands that do not enter into σ_t stacks, e.g. *releaser* and single strands bound into inert double helix waste products e.g. $\vdash pq \dashv$, $\vdash Zr \dashv$. Purity above $\mathcal{P} = 0.5$ indicates that the majority of the DNA strands in the system constitute the target stack species.

2. **[Xr]-[Yr]** is the nanomolar concentration difference between *X-read* and *Y-read* complexes in supernatant solution. It shows which signal is read out. Positive when [Xr] is in excess, negative when [Yr] is in excess, zero when the concentrations are equal and/or zero. Red dotted lines are used in Supplementary Figures 19 to 23 to show 10nM cutoffs for distinguishing concentrations.

3. **[Xr]+[Yr]** is the total nanomolar concentration of *X-read* and *Y-read* complexes in supernatant solution. It shows the absolute signal output value of the system.

4. **Yield** is the nanomolar concentration of the target stack species σ_t.

5. **Rank** is the order number of the target structure, if all structures in solution are placed in descending order by their molar concentrations. E.g. rank=1 if the target stack σ_t has the highest molar concentration of all stack species in solution; rank=2 if another stack species has a higher molar concentration than the target stack, and so on. **Rank (worst case)** is simply the highest rank of the target structure at each stage, over all stochastic simulation repeats for that stage. Here, "stack species" is defined as any species that is not *releaser*, and is not an inert double helix waste product. Rank is distinct from Purity: the target species σ_t may have rank 1 (highest molar concentration), but it may not command the majority of DNA strands in the system.

6. **#Species** is the number of qualitatively different molecular species existing in solution (not copy number, but species number). Note that this number pertains to the final state and does not include fleeting species created and then destroyed in the simulation phase.

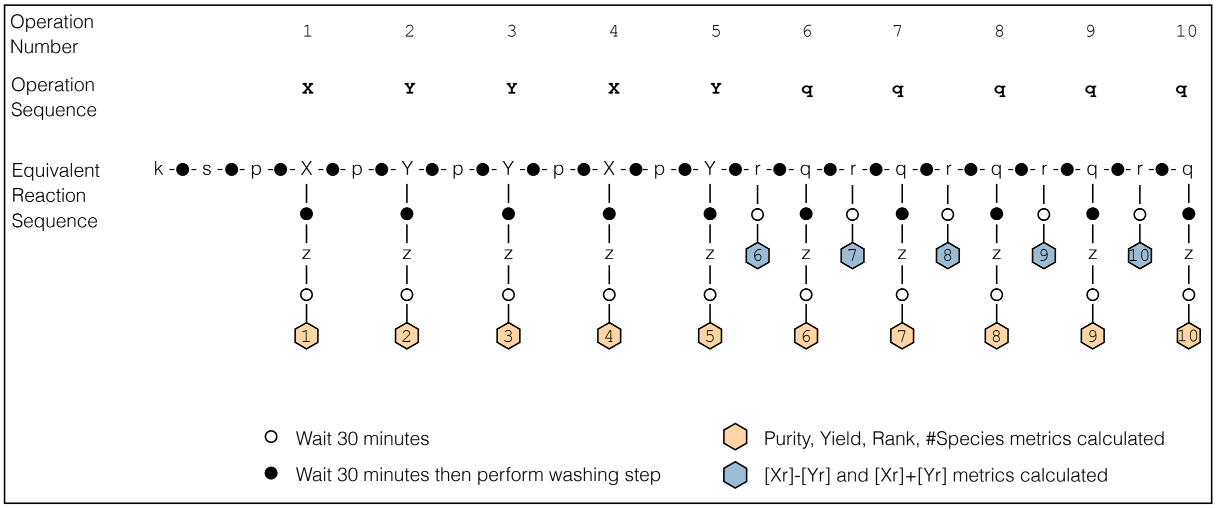

Supplementary Figure 18: Details of when stack metrics are calculated. Example operation sequence XYYXYqqqqq is expanded to the actual reaction sequence it is shorthand for. Branches off the reaction sequence show when/how the metrics are calculated at each operation. Numbers in the metric diamonds are the operation number the metric is reported at. Metrics Purity, Yield, Rank and #Species (yellow diamonds) concern the state of *stacks* in the system; they are therefore calculated after a full record or pop operation has been completed and *releaser* has been applied (releasing stacks on beads into the supernatant). Conversely, the [Xr]-[Yr] and [Xr]+[Yr] metrics (blue diamonds) concern the *X* and *Y* signals popped off stacks into supernatant. During a popping operation rq, they are calculated after *read* has been applied, when the *Xr* and *Yr* complexes are in the supernatant solution.

Supplementary Note 10.1.2 Results

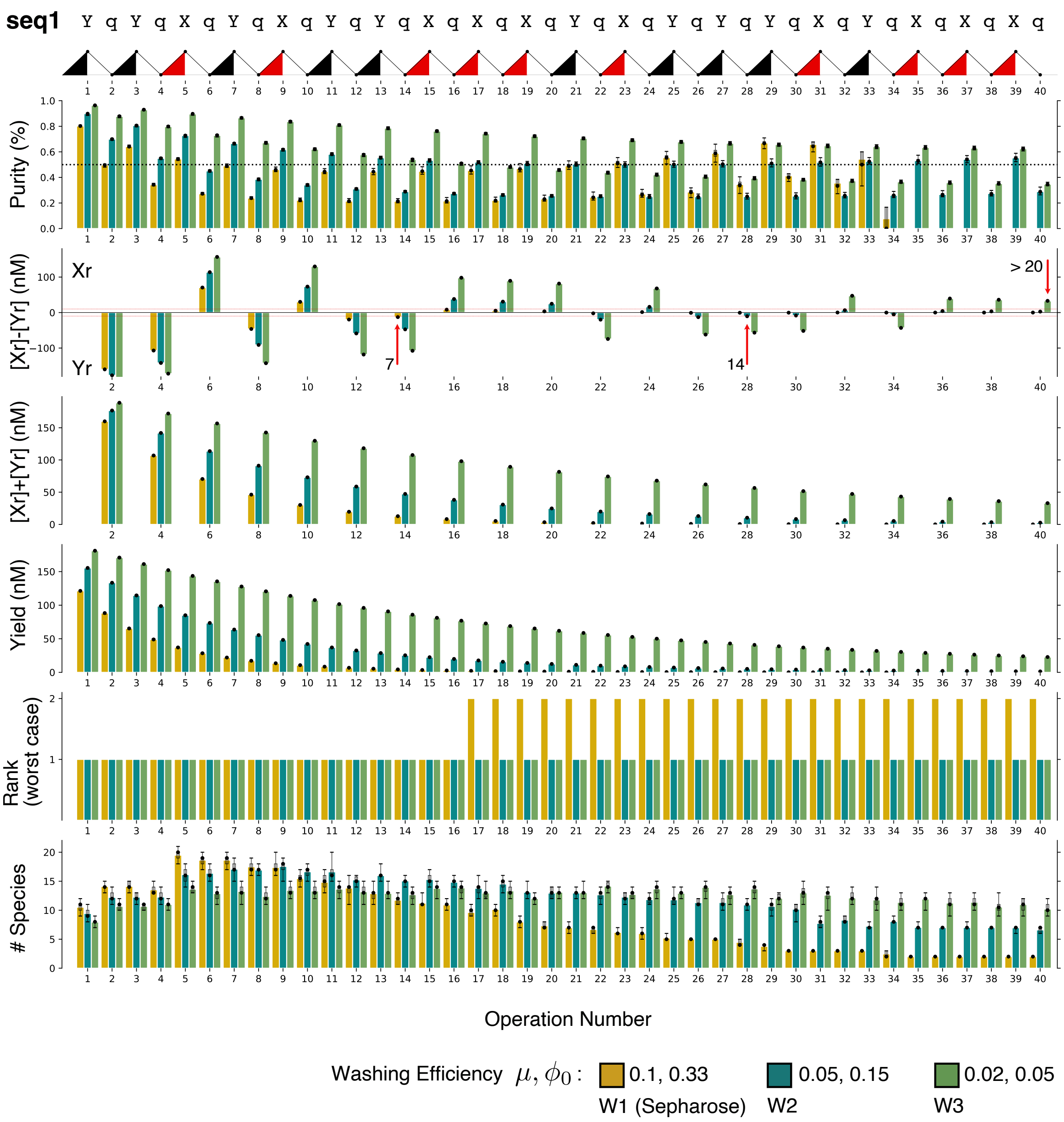

Supplementary Figure 19: *seq1:* step-by-step predicted performance of stack under W1, W2 and W3 washing efficiencies (each washing efficiency represented by a different coloured bar). Reaction wait time $t_w = 30$ minutes. Concentrations: *linker* = *releaser* = 200nM, all other strands $\lambda = 300$nM. See SI text for explanation of the 6 metrics used. Bar heights represent metric average values over $n = 50$ simulation repeats at each stage. Overlaid box and whisker plots show variance in the metrics: black dots indicate median values, boxes represents interquartile ranges (between 1st and 3rd quartiles) and whiskers represent minimum and maximum values over the $n = 50$ simulation repeats at each stage. The Rank (worst case) metric is an integer with no variance. Numbered red arrows on the [Xr]-[Yr] metric graph represent the maximum number of pops possible (the pop limit) at each washing efficiency.

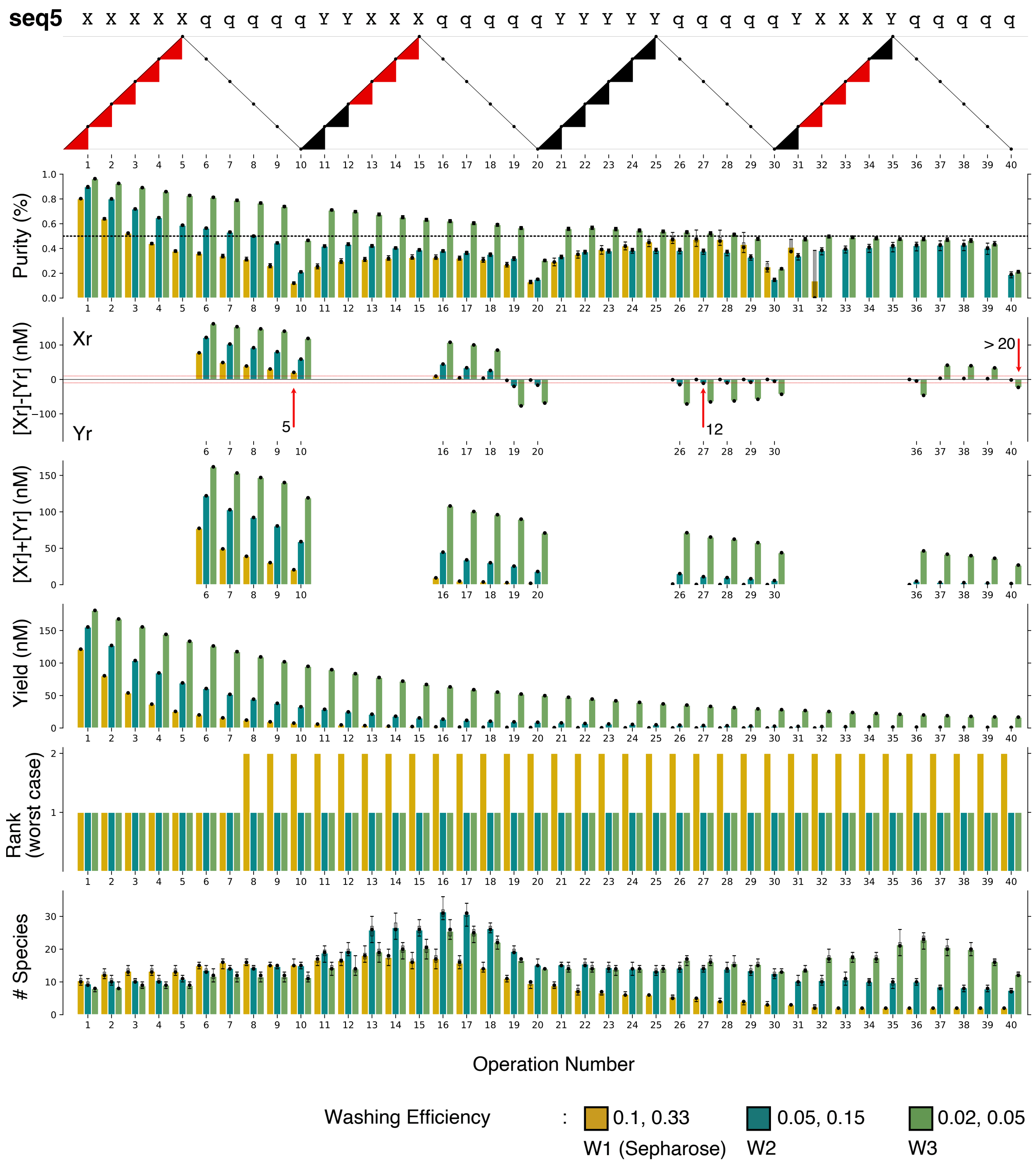

Supplementary Figure 20: *seq5:* step-by-step predicted performance of stack under W1, W2 and W3 washing efficiencies (each washing efficiency represented by a different coloured bar). Reaction wait time $t_w = 30$ minutes. Concentrations: *linker* = *releaser* = 200nM, all other strands $\lambda = 300$nM. See SI text for explanation of the 6 metrics used. Bar heights represent metric average values over $n = 50$ simulation repeats at each stage. Overlaid box and whisker plots show variance in the metrics: black dots indicate median values, boxes represents interquartile ranges (between 1st and 3rd quartiles) and whiskers represent minimum and maximum values over the $n = 50$ simulation repeats at each stage. The Rank (worst case) metric is an integer with no variance. Numbered red arrows on the [Xr]-[Yr] metric graph represent the maximum number of pops possible (the pop limit) at each washing efficiency.

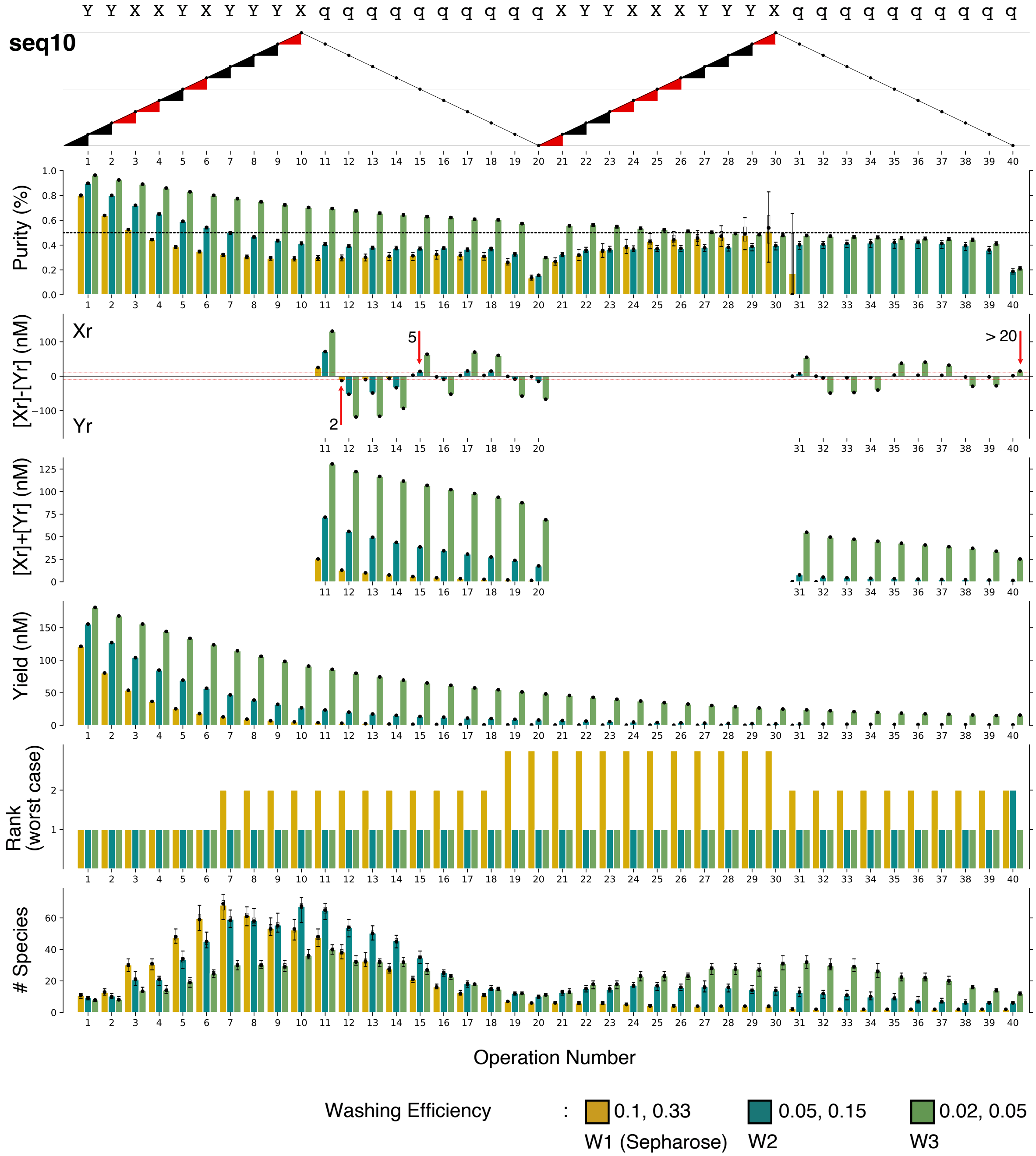

Supplementary Figure 21: *seq10:* step-by-step predicted performance of stack under W1, W2 and W3 washing efficiencies (each washing efficiency represented by a different coloured bar). Reaction wait time $t_w = 30$ minutes. Concentrations: *linker* = *releaser* = 200nM, all other strands $\lambda = 300$nM. See SI text for explanation of the 6 metrics used. Bar heights represent metric average values over $n = 50$ simulation repeats at each stage. Overlaid box and whisker plots show variance in the metrics: black dots indicate median values, boxes represents interquartile ranges (between 1st and 3rd quartiles) and whiskers represent minimum and maximum values over the $n = 50$ simulation repeats at each stage. The Rank (worst case) metric is an integer with no variance. Numbered red arrows on the [Xr]-[Yr] metric graph represent the maximum number of pops possible (the pop limit) at each washing efficiency.

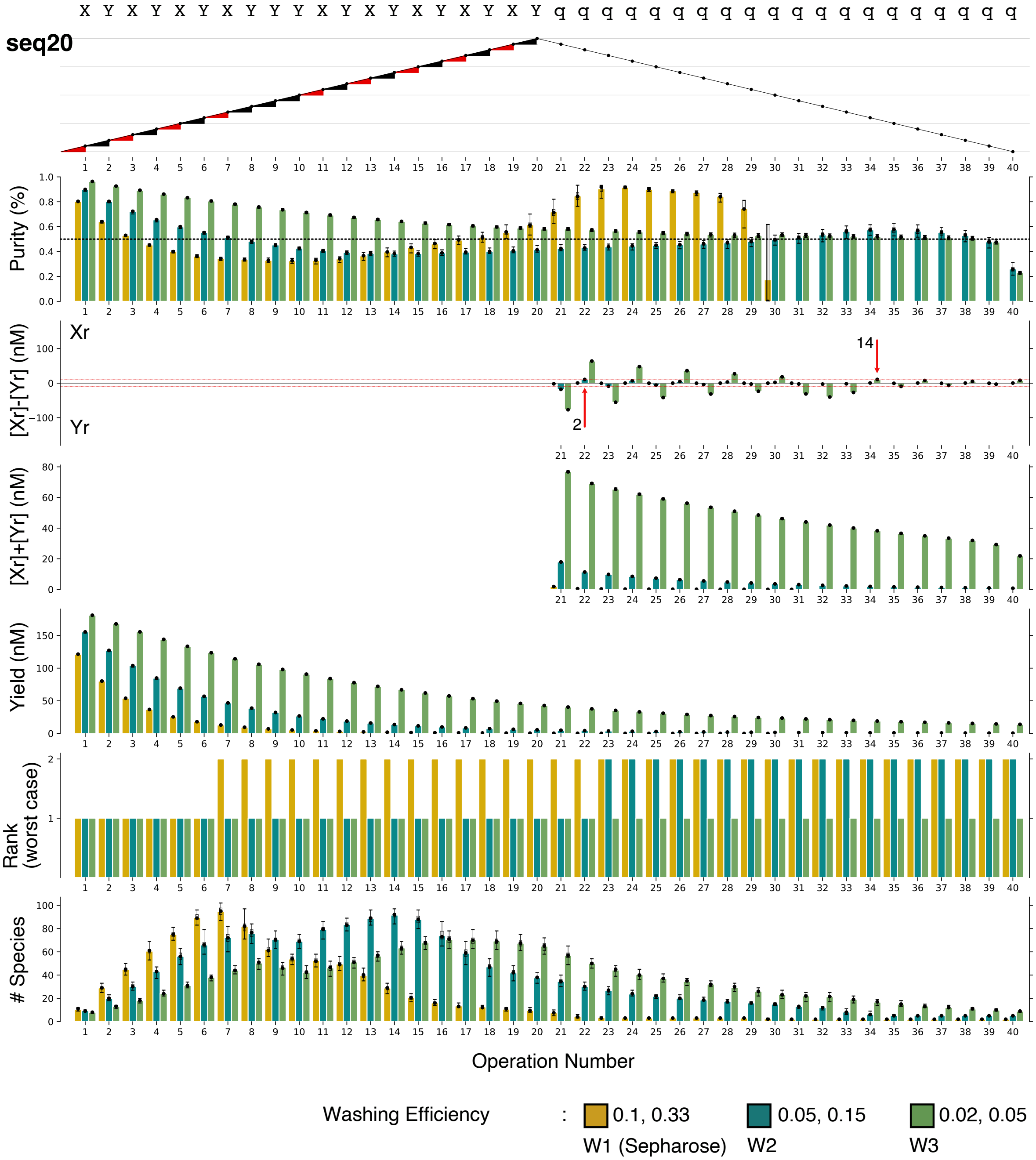

Supplementary Figure 22: *seq20:* step-by-step predicted performance of stack under W1, W2 and W3 washing efficiencies (each washing efficiency represented by a different coloured bar). Reaction wait time $t_w = 30$ minutes. Concentrations: *linker* = *releaser* = 200nM, all other strands $\lambda = 300$nM. See SI text for explanation of the 6 metrics used. Bar heights represent metric average values over $n = 50$ simulation repeats at each stage. Overlaid box and whisker plots show variance in the metrics: black dots indicate median values, boxes represents interquartile ranges (between 1st and 3rd quartiles) and whiskers represent minimum and maximum values over the $n = 50$ simulation repeats at each stage. The Rank (worst case) metric is an integer with no variance. Numbered red arrows on the [Xr]-[Yr] metric graph represent the maximum number of pops possible (the pop limit) at each washing efficiency.

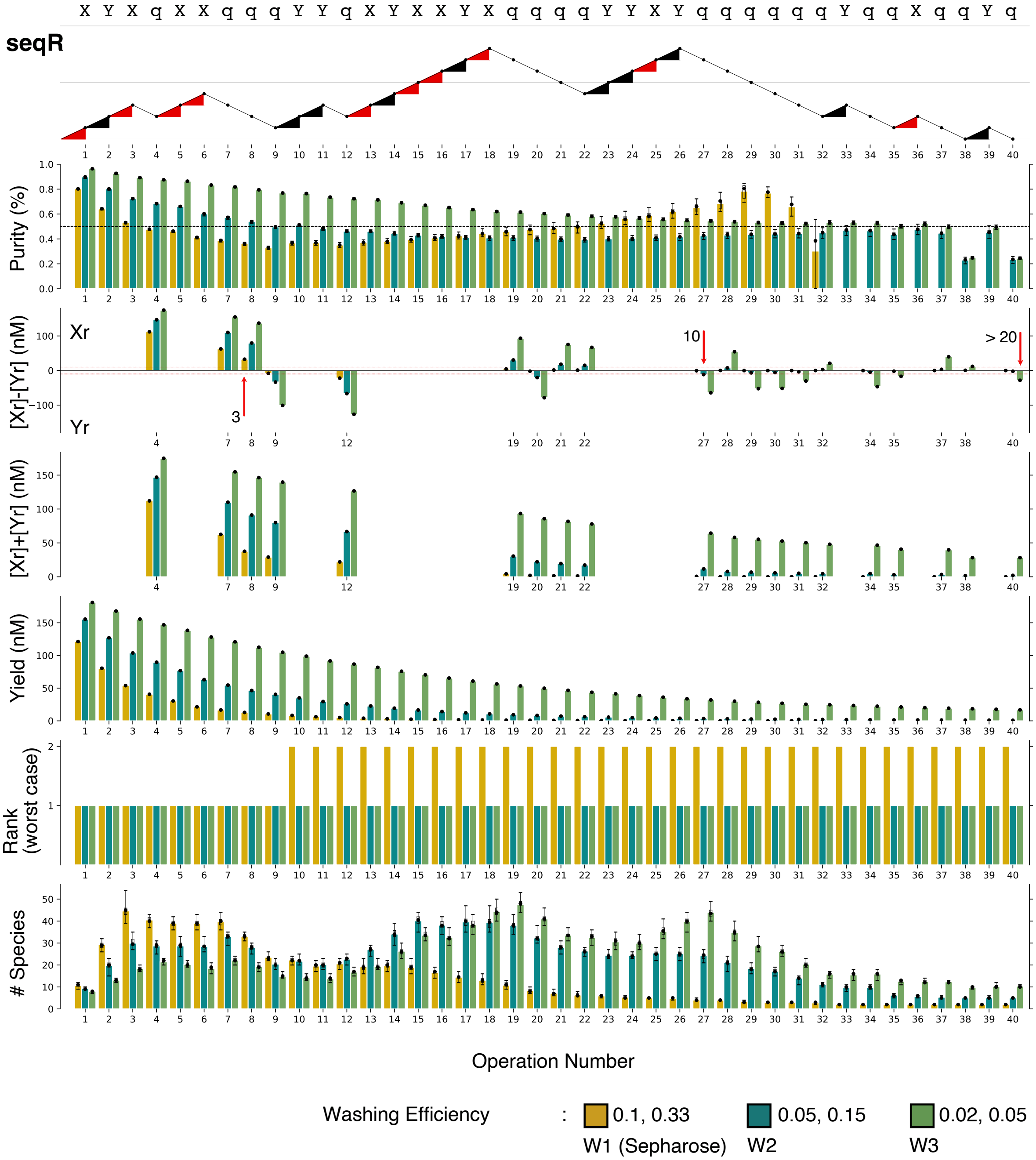

Supplementary Figure 23: *seqR:* step-by-step predicted performance of stack under W1, W2 and W3 washing efficiencies (each washing efficiency represented by a different coloured bar). Reaction wait time $t_w = 30$ minutes. Concentrations: *linker* = *releaser* = 200nM, all other strands $\lambda = 300$nM. See SI text for explanation of the 6 metrics used. Bar heights represent metric average values over $n = 50$ simulation repeats at each stage. Overlaid box and whisker plots show variance in the metrics: black dots indicate median values, boxes represents interquartile ranges (between 1st and 3rd quartiles) and whiskers represent minimum and maximum values over the $n = 50$ simulation repeats at each stage. The Rank (worst case) metric is an integer with no variance. Numbered red arrows on the [Xr]-[Yr] metric graph represent the maximum number of pops possible (the pop limit) at each washing efficiency.

Supplementary Note 10.1.3 Discussion

As can be seen in the step-by-step operation Supplementary Figures 19 to 23, for imperfect washing, target stack purity generally decreases below 50% before the majority signal popped into supernatant fails to be discerned (i.e. before |[Xr]-[Yr]| goes below the red dotted vertical lines of 10nM). As mentioned in the paper, this is due to the |[Xr]-[Yr]| metric only being dependent on the majority of stack ends in the system being the correct signal, whereas high purity requires the stronger condition that all stacks are identical. Target stack purity decreases very quickly for washing W1, falling to < 50% within just 4 operations for all operations sequences (i.e. the stack population quickly desynchronises). For some operations sequences (*seq1*, *seq20*, *seqR*), purity does appear to recover above 50% later on, but this is not a true recovery: rather, it is small system size artefact (purity is a *ratio*) when most beads have been washed out of the system and only a few stacks remain. The purity resurgence is indeed always followed by a sudden decrease to 0 purity, as the last beads (and stacks) are washed out.

Under W2 and W3 washings, the most concentrated stack species in the system (Rank = 1) also tends to be the target species. Thus, rank could also be used as a potential device read-out, but it is an open problem how the most concentrated Rank 1 stack species could be (i) identified and (ii) read by third party nucleic acid based circuits, particularly if only a small concentration difference existed between Rank 1 and Rank 2, or if very low concentrations are involved.

Thus, majority signal popped into supernatant emerges as the most robust and most practical read out of the stack system. Supplementary Figures 19 to 23 show that the reason the majority popped signal eventually becomes indistinguishable (i.e. the popped signal complexes *Xr* and *Yr* are not separated by at least 10nM concentration) is because the total signals popped into supernatant ([Xr]+[Yr]) become 0 over time. This is due to bead loss removing stack species from the system over time.

The #Species metric shows that stack population diversity increases during prolonged recording phases and decreases during prolonged popping phases (for W2 and W3 where bead loss is not also a complicit factor in reducing population diversity). Diversity decrease during popping is partially due to the truncation of stacks to lower bound size (i.e. just *start*), below which no further truncation can happen.

Supplementary Note 10.2 Pop Limit Sensitivity to Washing Procedure Efficiency

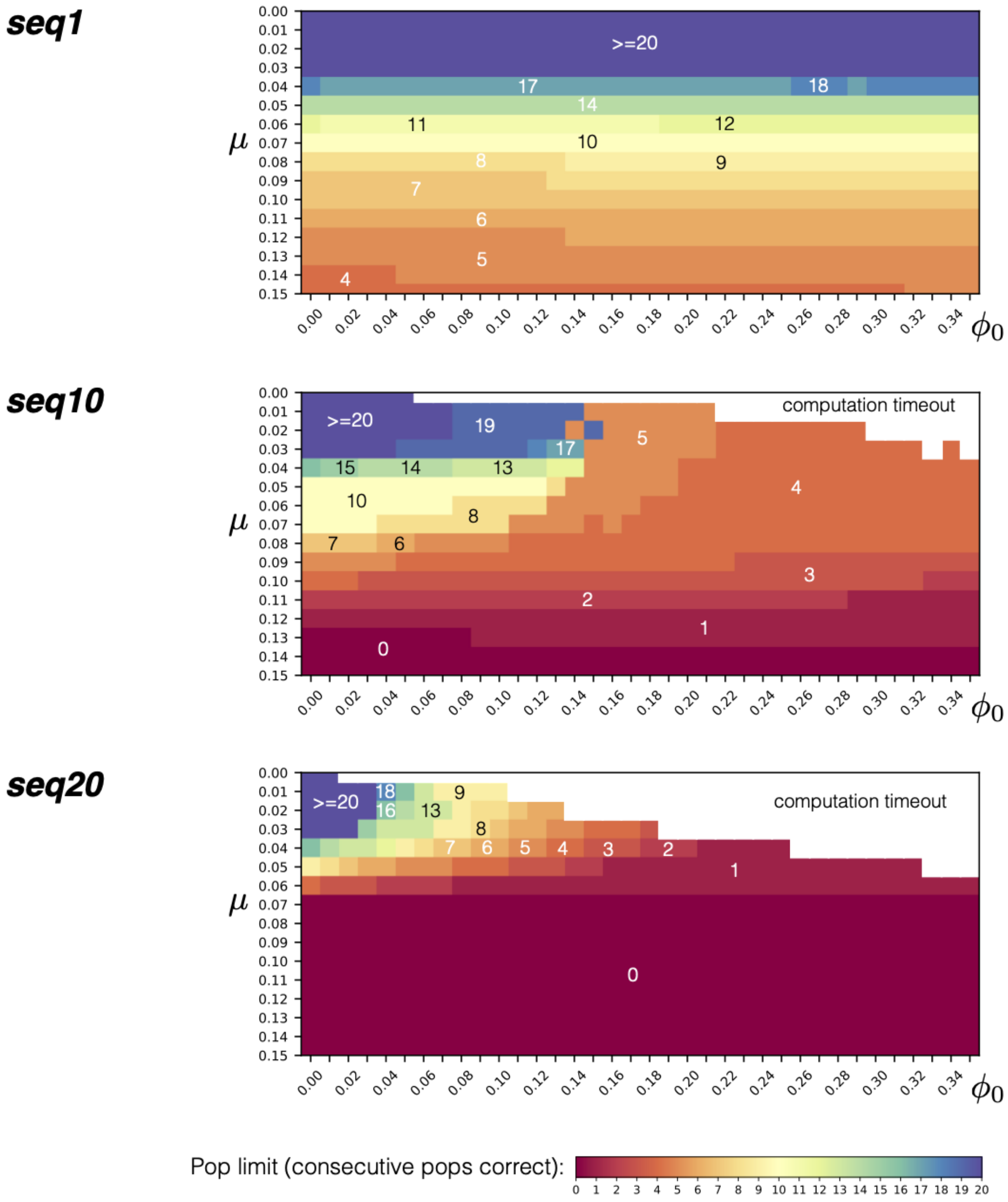

Supplementary Figure 24: How pop limit depends on efficiency of washing procedure (μ and ϕ_0) for *seq1*, *seq10* and *seq20*. Each heatmap grid square derived from a single simulation. White squares signify stochastic simulation did not complete after 30 hours wall time. Strand concentrations $\lambda = 300nM$, reaction wait time is 30 minutes. Extends data in Figure 3 of main paper.

Supplementary Note 10.3 Pop Limit Sensitivity to Concentrations, Wait Times, Rate Constants

Model predictions of how adjusting a) the concentration λ of all strands (except *linker* and *releaser* constant at 200nM) and b) the reaction wait time t_w affect stack performance for all operations sequences under W1, W2 and W3 washing regimes. Extends data in Figure 3 of main paper.

Additionally, the effect of changing hybridisation constants k_A and k_{BC} is explored for washing regime W2. To extend the existing performance range both k_A and k_{BC} rate constants must be made faster, otherwise one rate limits the other (since stack polymer elongation and truncation always involves alternating these two reactions).

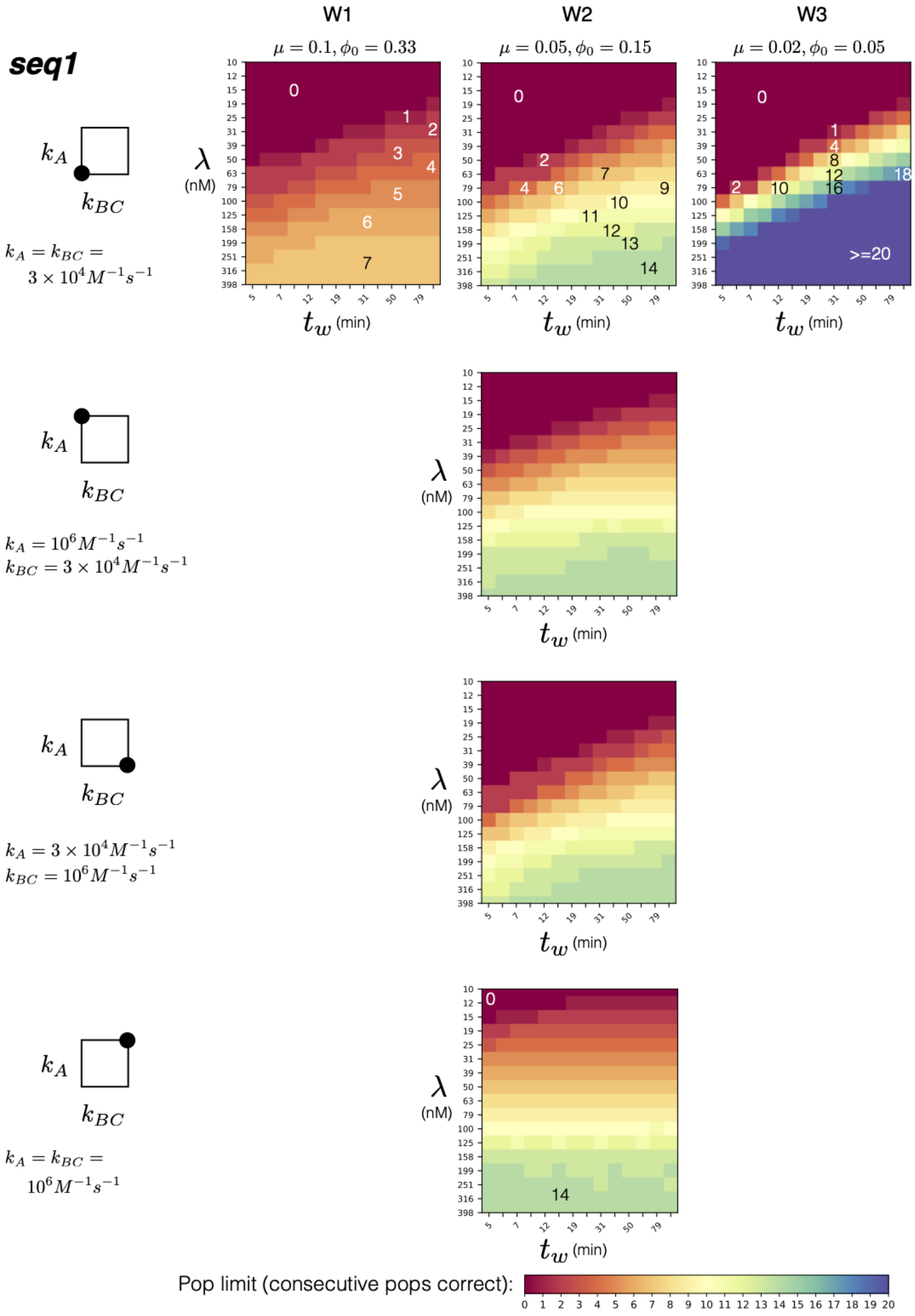

Supplementary Figure 25: Operations sequence *seq1*: dependence of pop limit on strand concentrations and wait times (λ and t_w), for the three washing scenarios W1, W2, W3. Further dependence on rate constants is shown for W2. Each heatmap grid square derived from a single simulation.

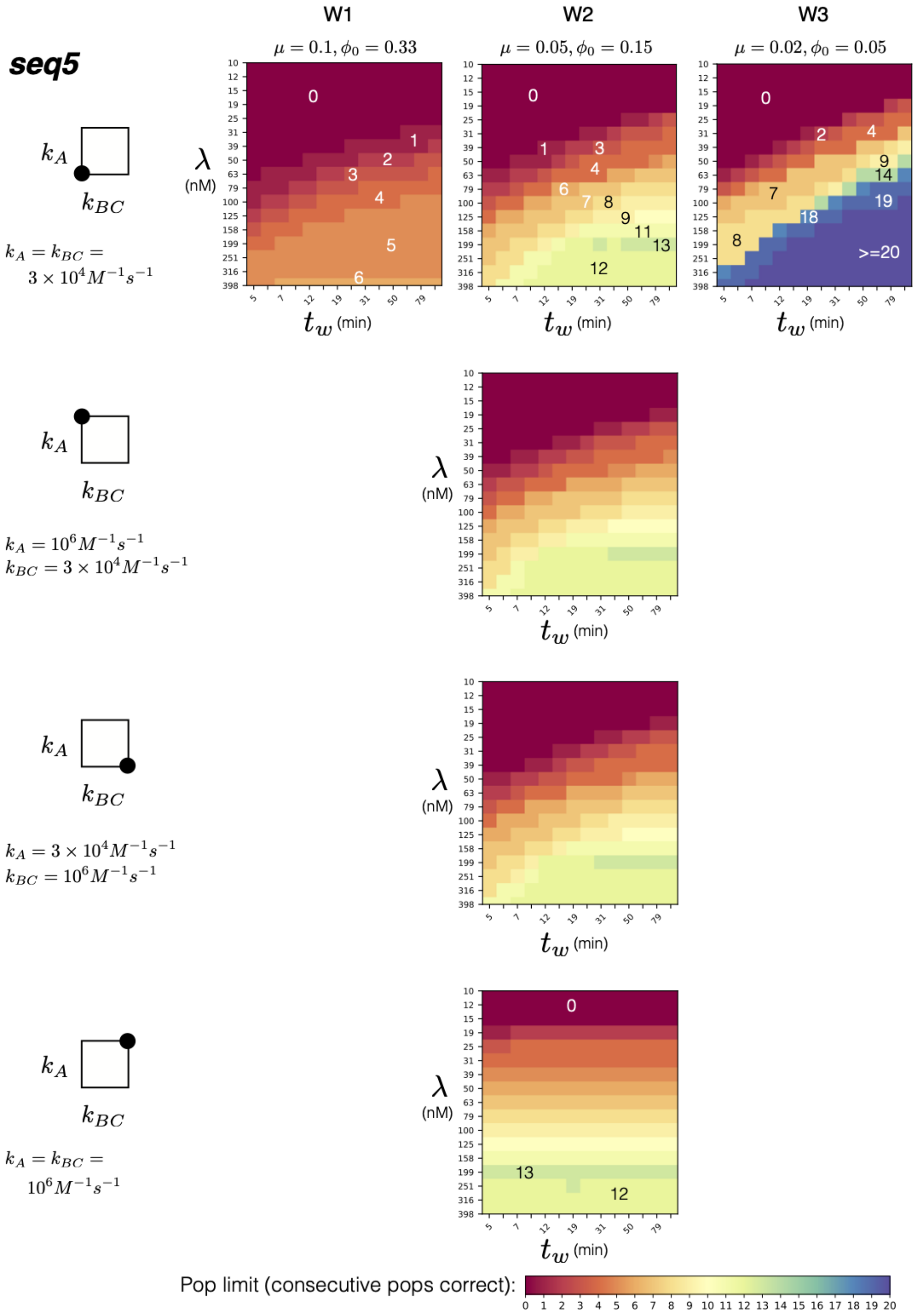

Supplementary Figure 26: Operations sequence *seq5*: dependence of pop limit on strand concentrations and wait times (λ and t_w), for the three washing scenarios W1, W2, W3. Further dependence on rate constants is shown for W2. Each heatmap grid square derived from a single simulation.

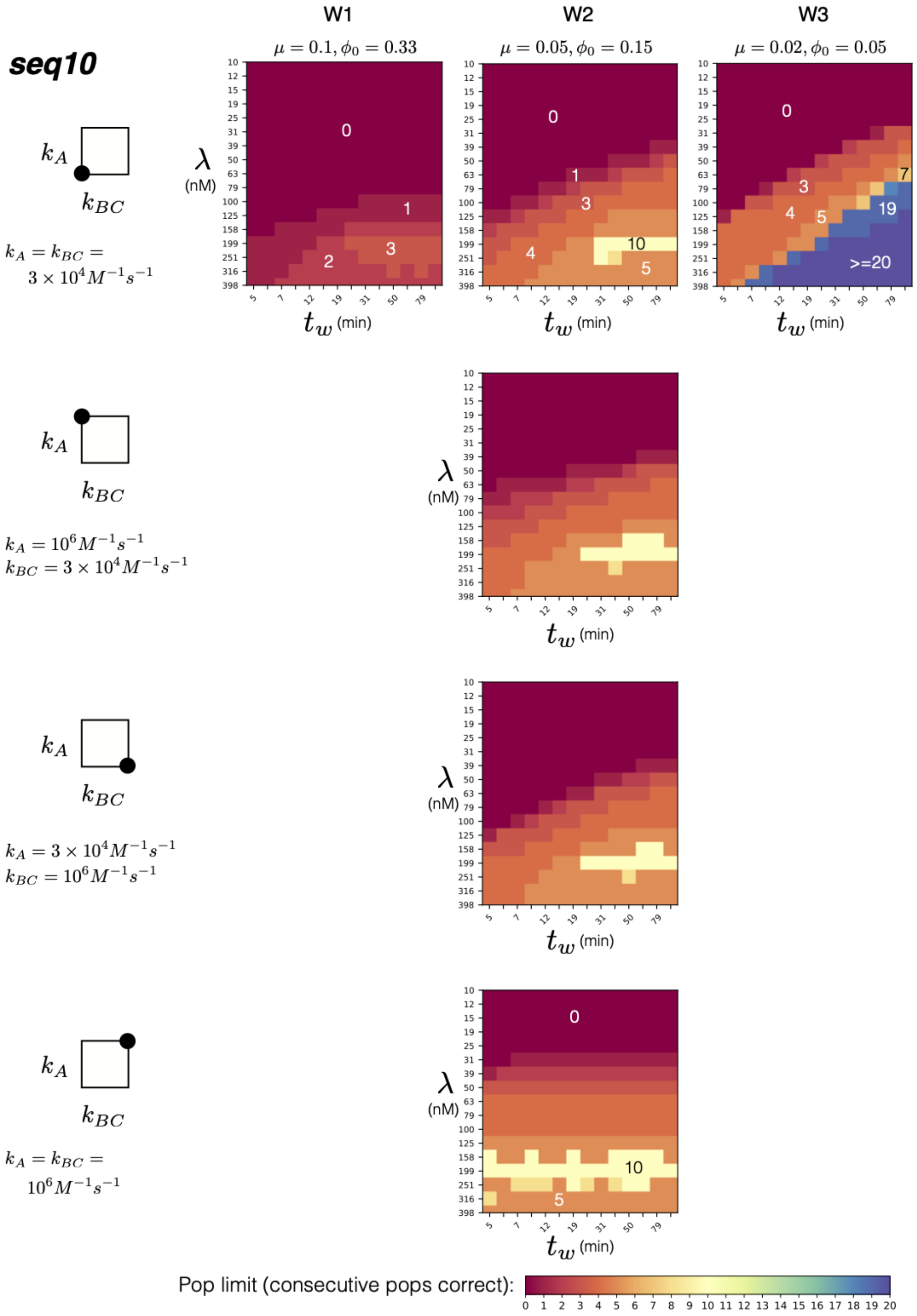

Supplementary Figure 27: Operations sequence *seq10*: dependence of pop limit on strand concentrations and wait times (λ and t_w), for the three washing scenarios W1, W2, W3. Further dependence on rate constants is shown for W2. Each heatmap grid square derived from a single simulation.

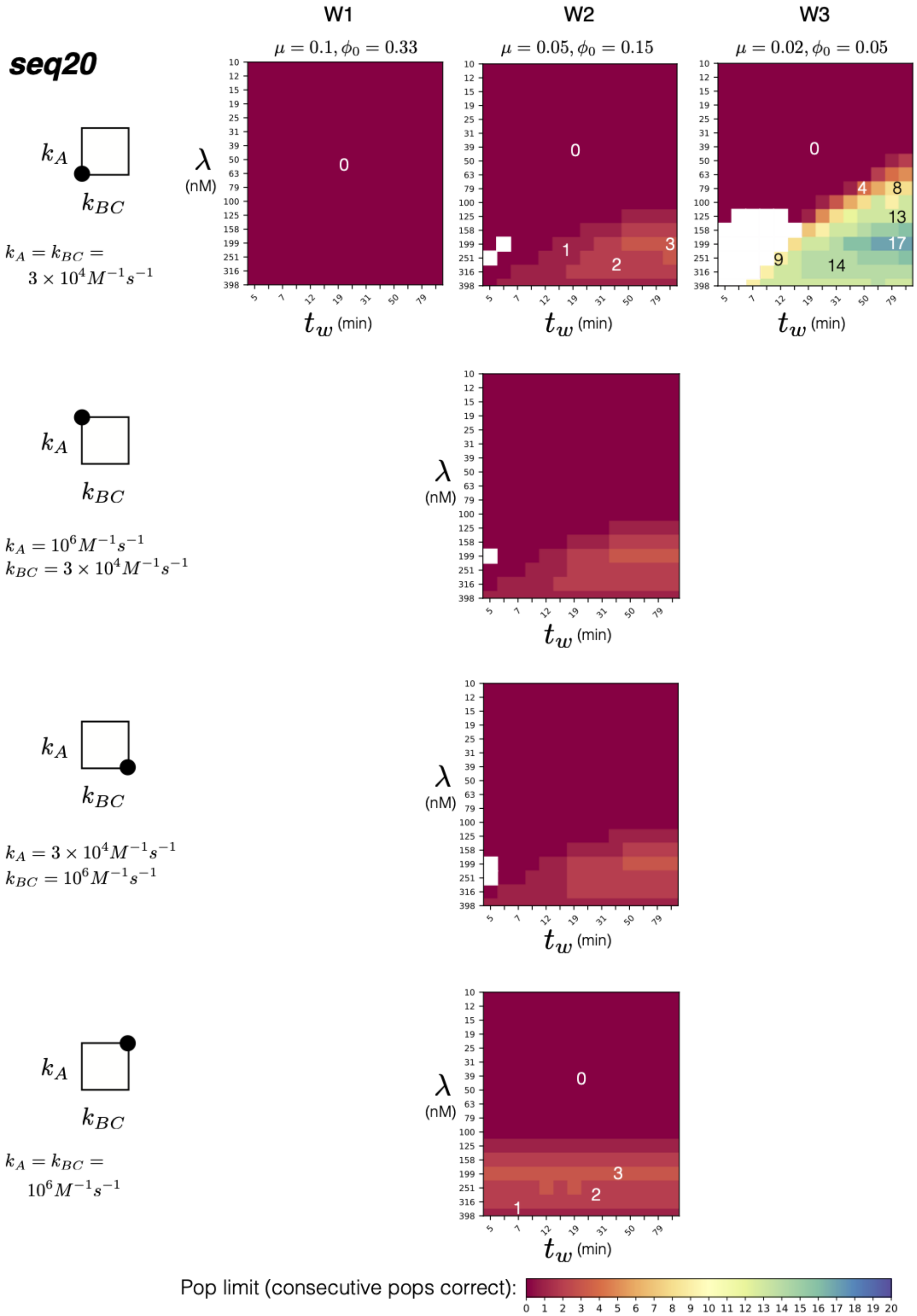

Supplementary Figure 28: Operations sequence *seq20*: dependence of pop limit on strand concentrations and wait times (λ and t_w), for the three washing scenarios W1, W2, W3. Further dependence on rate constants is shown for W2. Each heatmap grid square derived from a single simulation. White squares signify stochastic simulation did not complete after 30 hours wall time.

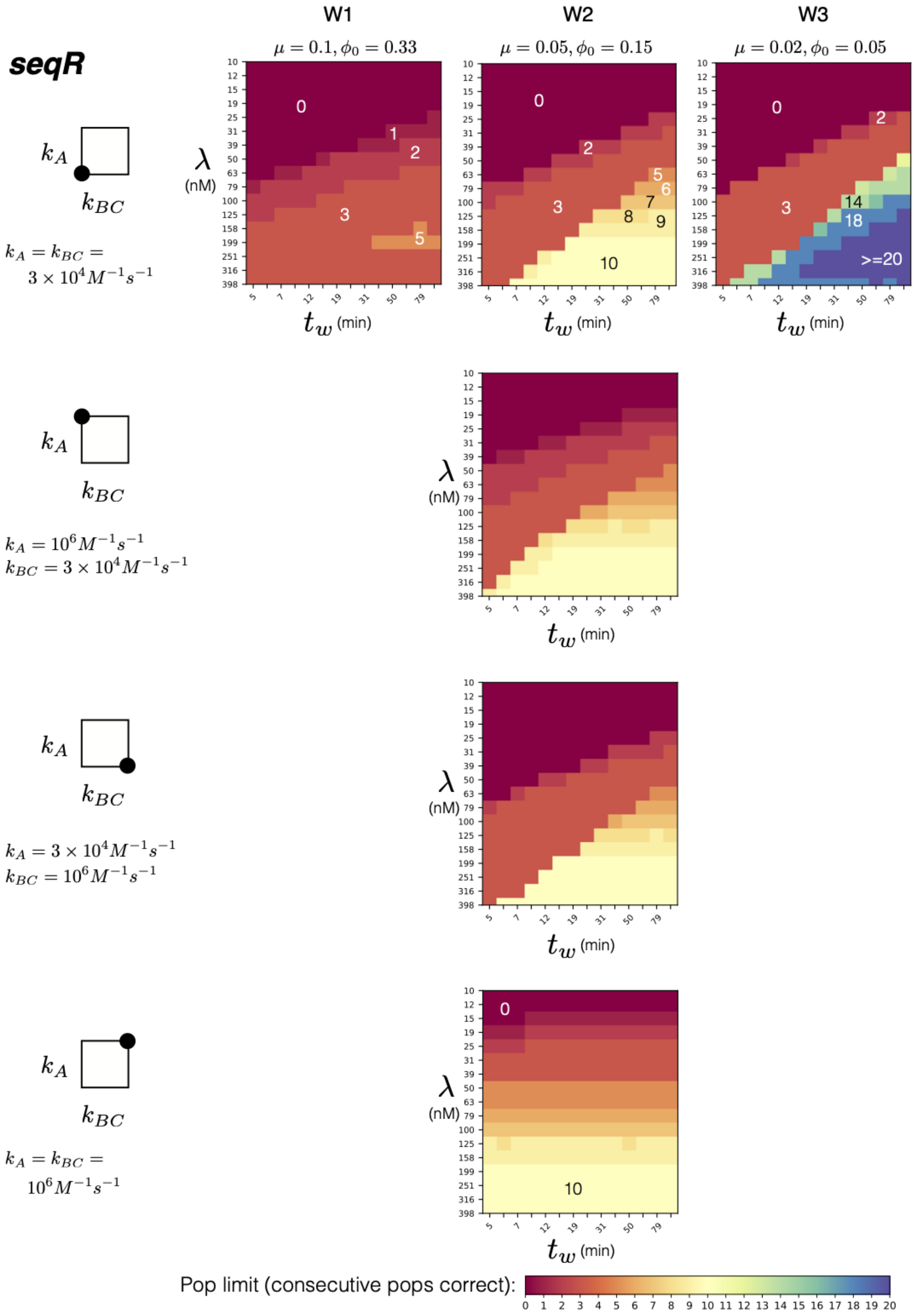

Supplementary Figure 29: Operations sequence *seqR*: dependence of pop limit on strand concentrations and wait times (λ and t_w), for the three washing scenarios W1, W2, W3. Further dependence on rate constants is shown for W2. Each heatmap grid square derived from a single simulation.

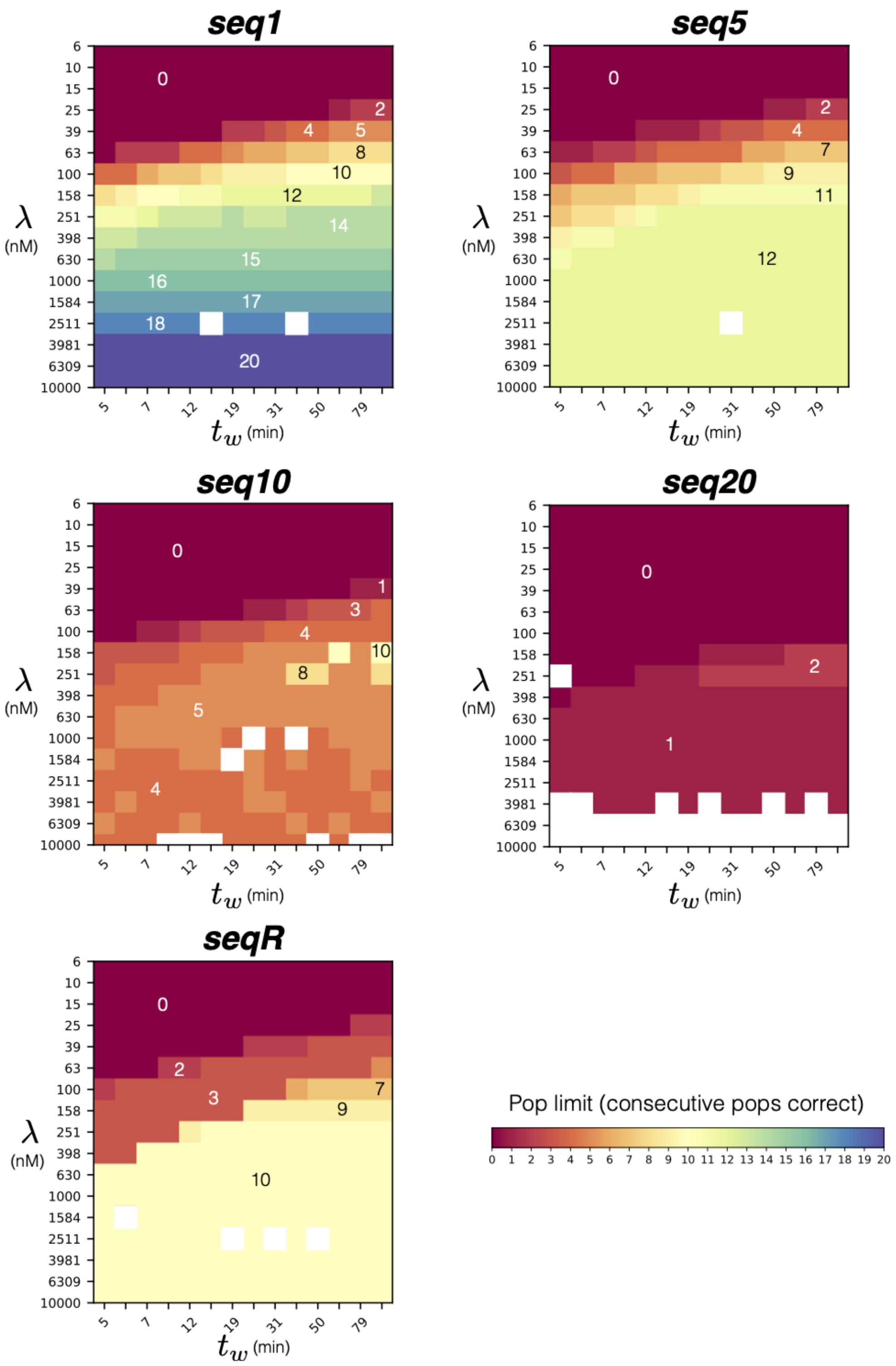

Supplementary Figure 30: Effect of high concentration strands. Dependence of pop limit on strand concentrations and wait times (λ and t_w) where strand concentrations are extended to 10μM under washing efficiency W2 ($\mu = 0.05, \phi_0 = 0.15$). Each heatmap grid square derived from a single simulation. White squares signify stochastic simulation did not complete after 30 hours wall time. *seq1* is the only operations sequence improving performance at high strand concentrations due to its pathological structure: each signal recorded is immediately read on the next step. Hence, a large supernatant excess of the last signal transferred to the next step via non-specific bead binding helps read out the correct signal, even when beads are diminishing.

Supplementary Note 10.4 Pop Limit Sensitivity to Pipetting Noise

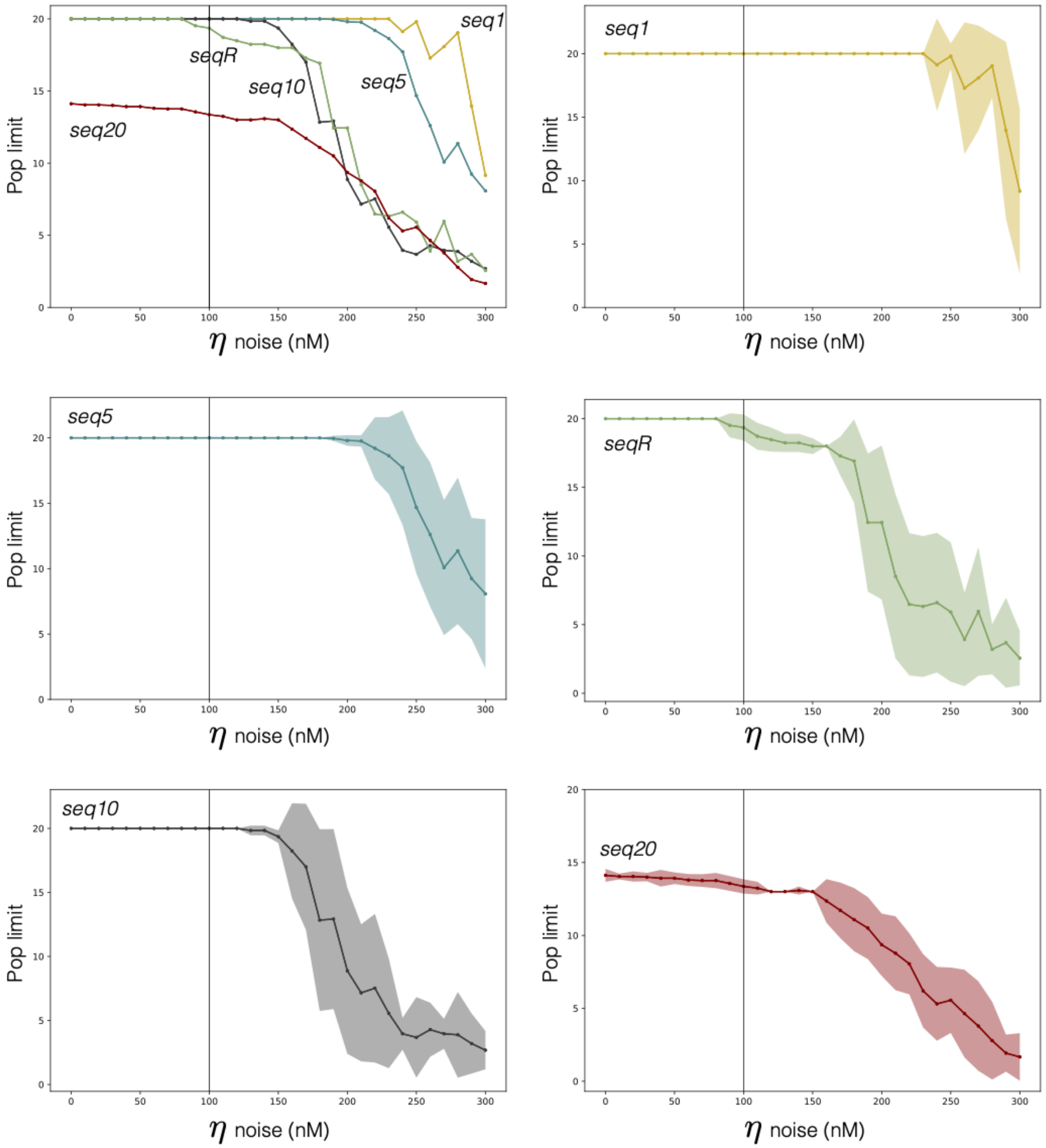

Supplementary Figure 31: Average pop limit (lines) for all operations sequences under W3 washing, also showing all standard deviation shadows on separate plots. Each standard deviation calculated from $n = 25$ independent simulation repeats. Extends data in Figure 3g of main paper. Beyond $\eta > 180$nM, not all $n = 25$ simulation repeats of *seq20* completed within 30 hours wall time: in the worst case, $n = 9$ repeats were used to estimate the standard deviation.

Supplementary Note 10.5 Model Prediction of Experiments with Looped Signals *X* and *Y*

Supplementary Note 10.5.1 Bioanalyzer Experiments in Figure 4c,d Main Paper

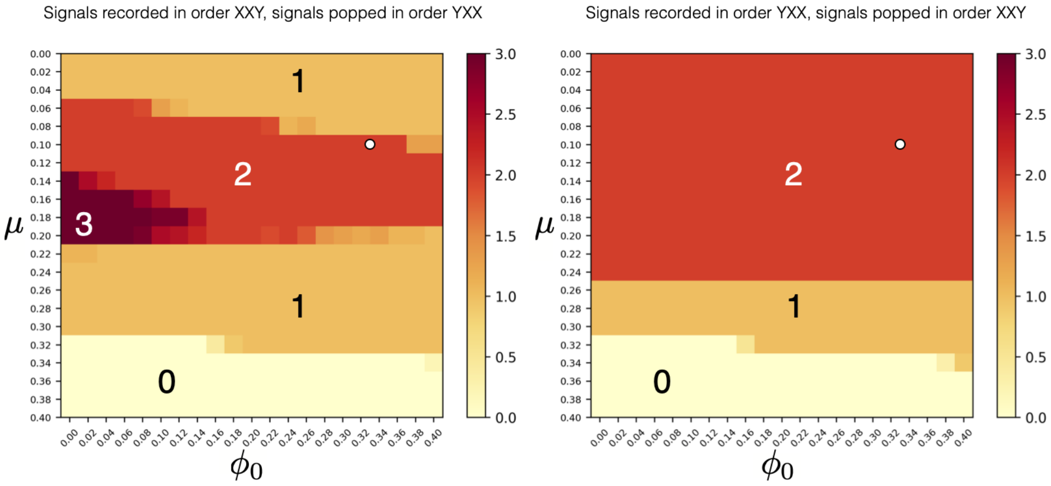

Read 50nM, *linker* and *releaser* 200nM, all other strand concentrations 300nM.

Supplementary Figure 32: *Read* at reduced 50nM concentration. Model prediction of the average ($n = 10$ repeats) number of signals popped successfully for XXY recorded (left, for experiment in Figure 4c of main paper) and YXX recorded (right, for experiment in Fig 4d main paper), over a range of washing efficiencies. Washing efficiency W1 ($\mu = 0.1,\ \phi_0 = 0.33$) for our experimental setup (white dots) are predicted to yield 2 signals successfully popped. The washing parameter region giving 3 successful pops is very narrow or absent altogether. In the model (where impurities are not present), *read* at reduced concentration leads to worse performance because it does not pop signals from all stacks and thus population diversity increases with each popped signal.

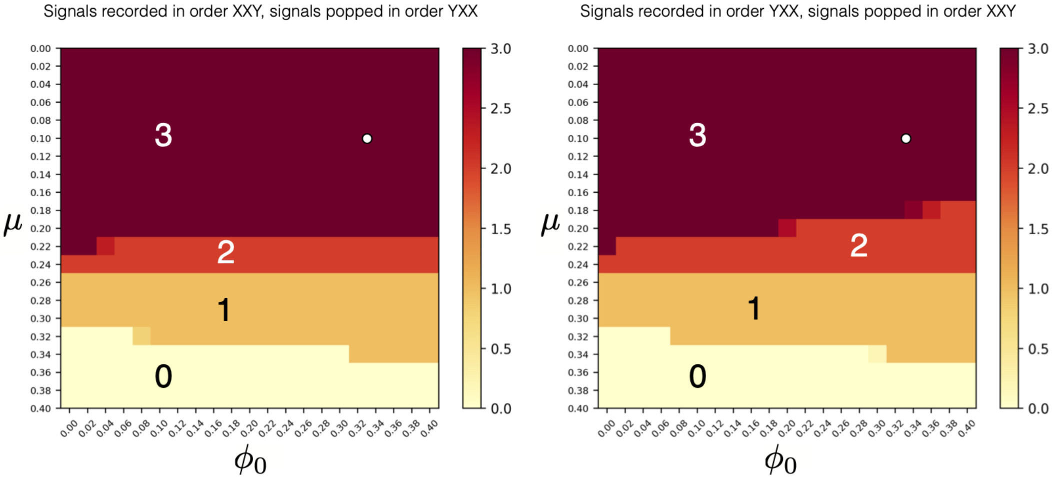

Linker and *releaser* 200nM. All other strand concentrations 300nM.

Supplementary Figure 33: *Read* at 300nM. Model prediction of the average ($n = 10$ repeats) number of signals popped successfully for XXY recorded (left) and YXX recorded (right). Washing efficiency W1 ($\mu = 0.1,\ \phi_0 = 0.33$) for our experimental setup (white dots) should reliably yield 3 signals successfully popped.

Supplementary Note 10.5.2 AFM Micrographs in Figure 4g Main Paper and Supplementary Note 6

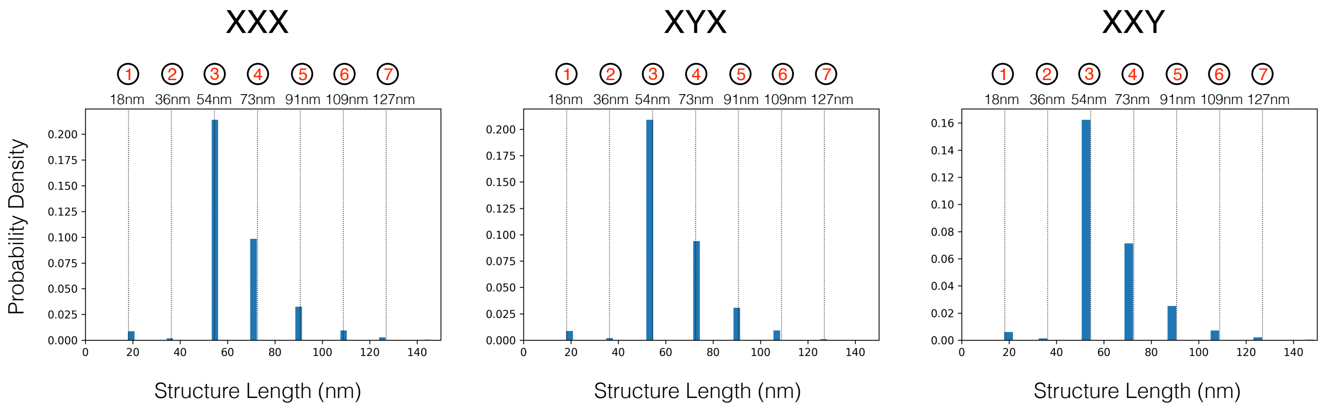

Supplementary Figure 34: Model prediction of AFM size distributions for 3 signal recording experiments for washing efficiency W1 ($\mu = 0.1,\ \phi_0 = 0.33$). Prediction "XXX" corresponds to Figure 4g in main paper. Predictions "XYX" and "XXY" correspond to Supplementary Figure 12. Circles with numbers N denote where stack complexes recording N signals lie on the end-to-end structure size scale. The model forecasts that 3 signal recording experiments XXX, XYX and XXY will result in a majority of 3-signal stacks, with higher order stacks (4,5,6,7 signals) present in exponentially decreasing amounts. It should be noted that multiple factors can affect the size distribution obtained from AFM images, e.g. fragmentation during deposition on mica and disruption by AFM probes during AFM imaging [7, 8]. But, notably, the model only predicts trace quantities of 2-signal stacks whereas structures of length 2 signals are the *most common* in the experimental AFM micrographs (and are also present in the Bioanalyzer analyses of the samples). Conversely, PAGE analysis of recording 3 linear *write* strands (e.g. Supplementary Figure 1 lane 5, Supplementary Figure 3 lane 7) showed only trace amounts of 2-signal stacks, in closer agreement with the model.

Supplementary Note 11 Oligonucleotide Synthesis Purity

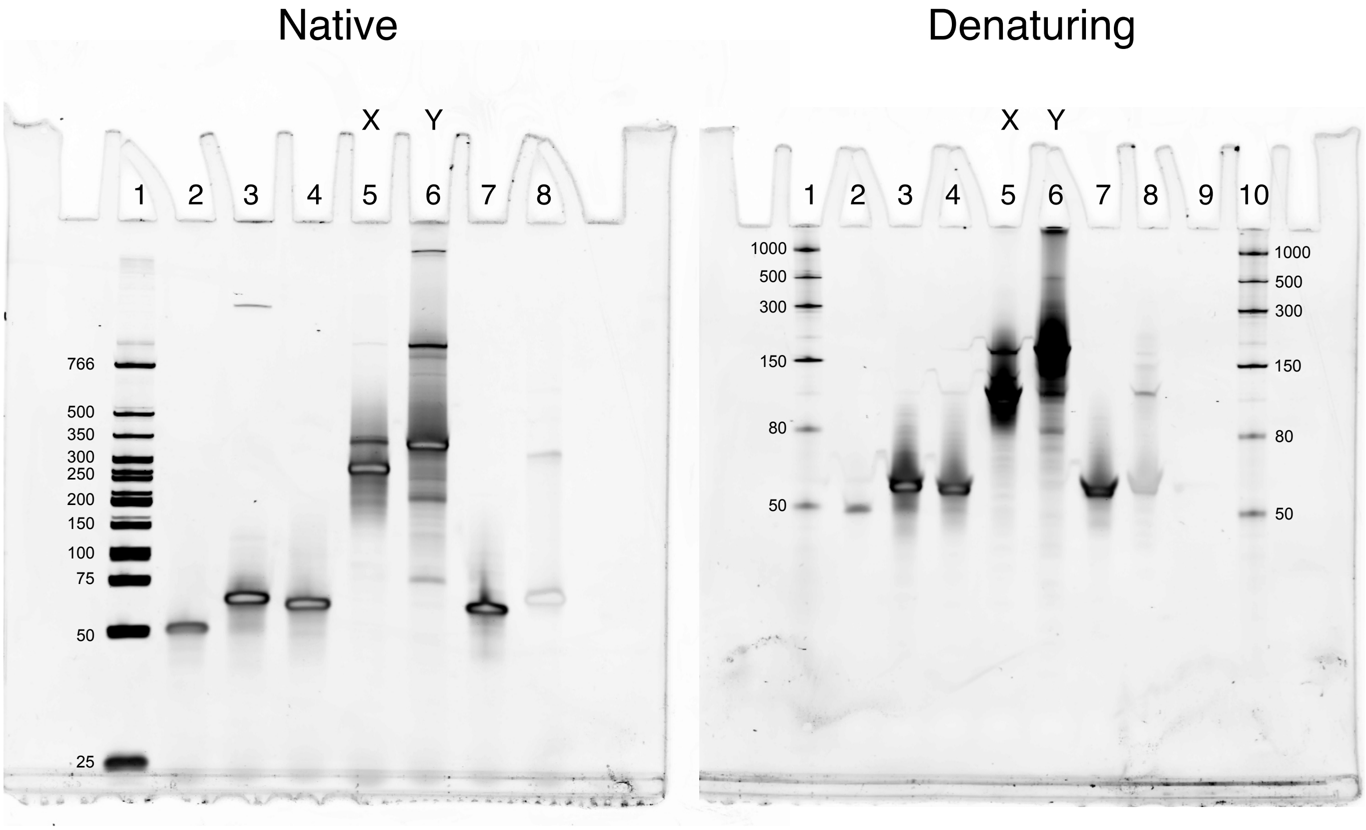

Supplementary Figure 35: Purity of chemically synthesized oligonucleotides used for stack assembly. TBE gel electrophoresis of single strands *start* (lane 2), *push* (3), *write* (4), *X* (5), *Y* (6), *pop* (7) and *p-glow* (8), under native (left: 10% TBE gel) and denaturing conditions (right: 10% denaturing TBE-Urea). Denaturing analysis confirms off-target bands are distinct mis-synthesised ssDNA fragments rather than alternate secondary structure folds of the target strand. For 10% denaturing TBE-Urea gel, samples were denatured at 90 °C for 5 min and incubated in ice for 2 min. After staining with SYBR® Gold in 1x TBE for 5 min, the gels were visualized using Typhoon laser scanner (normal sensitivity, PMT 376 or 600 V, Cy3 channel) and ImageQuant TL software (GE Healthcare Life Sciences). Low molecular weight DNA ladder (molecular weight markers in base pairs) and low range ssRNA ladder (molecular weight markers in bases) were used for native and denaturing gels respectively (both from NEB). Mass of strands used: *start* (115 ng), *push* (130 ng), *write* (130 ng), *X* (247 ng), *Y* (316 ng), *pop* (130 ng), *p-glow* (130 ng). The latter quantities (in ng) are loaded considering the mass of respective strands in 100 nM reaction mixture (total volume 70 uL). This purity control was run a single time.

References

[1] Kozyra, J., Fellermann, H., Shirt-Ediss, B., Lopiccolo, A. & Krasnogor, N. Optimizing nucleic acid sequences for a molecular data recorder. In *GECCO 2017 - Proceedings of the 2017 Genetic and Evolutionary Computation Conference* (2017).

[2] Shin, J.-S. & Pierce, N. A. Rewritable Memory by Controllable Nanopatterning of DNA. *Nano Letters* **4**, 905–909 (2004).

[3] Bielec, K. *et al.* Kinetics and equilibrium constants of oligonucleotides at low concentrations. Hybridization and melting study. *Physical Chemistry Chemical Physics* **21**, 10798–10807 (2019).

[4] Wetmur, J. G. & Davidson, N. Kinetics of renaturation of DNA. *Journal of Molecular Biology* **31**, 349–370 (1968).

[5] Srinivas, N. *et al.* On the biophysics and kinetics of toehold-mediated DNA strand displacement. *Nucleic Acids Research* **41**, 10641–10658 (2013).

[6] Zhang, D. Y. & Winfree, E. Control of DNA strand displacement kinetics using toehold exchange. *Journal of the American Chemical Society* **131**, 17303–14 (2009).

[7] Li, M. *et al.* In vivo production of RNA nanostructures via programmed folding of single-stranded RNAs. *Nature Communications* **9**, 2196 (2018).

[8] Wei, B., Dai, M. & Yin, P. Complex shapes self-assembled from single-stranded DNA tiles. *Nature* **485**, 623–626 (2012).
